# Metastasis-directed SBRT for oligometastatic hormone sensitive prostate cancer (METRO): protocol for a prospective randomised phase III trial, NCT04983095

**DOI:** 10.1186/s12885-026-15906-6

**Published:** 2026-03-25

**Authors:** Karin Söderkvist, Maira Zia, Adalsteinn Gunnlaugsson, Andreas Josefsson, Bjørg Aksnessæther, Chunde Li, Camilla Thellenberg-Karlsson, Daniel Alm, David Kudrén, Erik Lundin, Gabriel Moise, Jenny Kahlmeter Brandell, Jon Kindblom, Kirsten Björnlinger, Kristin Karlsson, Katrine Riklund, Martina Westin, Mattias Hedman, Natalie Skorve, Pernilla Wikström, Sara Strandberg, Joakim Jonsson

**Affiliations:** 1 Department of Diagnostics and Intervention, Umeå University, Umeå, Sweden; 2 Hematology, Oncology and Radiation Physics, Skåne University Hospital, Lund University, Lund, Sweden; 3 Wallenberg Center for Molecular Medicine, Umeå University, Umeå, Sweden; 4 Department for Cancer and Rehabilitation, Ålesund Hospitalt, Helse Møre and Romsdal Hospital Trust, Ålesund, Norway; 5 Clinical Science and Education, Karolinska Institutet, Stockholm, Sweden; 6 Oncology, Stockholm South General Hospital, Stockholm, Sweden; 7 Radiation Oncology, Karolinska University Hospital, Stockholm, Sweden; 8 Oncology, Örebro University Hospital, Örebro, Sweden; 9 Oncology, County Hospital Ryhov, Jönköping, Sweden; 10 Oncology, Clinical Sciences, Sahlgrenska Academy at Gothenburg University, Gothenburg, Sweden; 11 Radiotherapy Physics and Engineering, Karolinska University Hospital, Stockholm, Sweden; 12 Oncology-Pathology, Karolinska Institute, Stockholm, Sweden; 13 OncologyCapio Saint Göran’s Hospital, Stockholm, Sweden; 14 Department for Clinical and Molecular Medicin, NTNU, St Olavs Hospital, Trondheim, Norway; 15 Department of Medical Biosciences, Umeå University, Umeå, Sweden

**Keywords:** Oligo-metastatic, Hormone sensitive prostate cancer, Stereotactic body radiotherapy, Phase III randomised controlled trial, Study protocol

## Abstract

**Background:**

Metastasis-directed stereotactic body radiotherapy (MD-SBRT) has shown promise in retrospective and phase II studies for oligometastatic hormone-sensitive prostate cancer. However, prospective randomized phase III data—particularly in newly diagnosed cases and in combination with androgen deprivation therapy and next-generation androgen receptor pathway inhibitors—are limited. The METRO trial investigates the addition of MD-SBRT to standard of care in patients with prostate-specific membrane antigen (PSMA) PET/CT-detected oligometastatic disease.

**Methods:**

METRO is a multicentre, double arm, open-label, phase III randomized trial comparing MD-SBRT plus standard of care versus standard of care alone in patients with one to three PSMA PET/CT-detected distant metastases. The PSMA-RADS scale is used to support inclusion, and only patients with PSMA-RADS 4 or 5 lesions in bone or non-regional lymph nodes are eligible.

Standard of care includes time-limited androgen deprivation therapy and/or androgen receptor pathway inhibitor, as well as local radiotherapy to the prostate or prostate bed. Patients are stratified by disease type (synchronous or metachronous) and metastasis location (lymph node/bone). The primary endpoint is biochemical progression-free survival; secondary endpoints include time to castration-resistant prostate cancer, adverse events, and health-related quality of life.

The intervention is prescribed either 30 Gy in 3 fractions or 40 Gy in 5 fractions and delivered by stereotactic treatment principles.

**Discussion:**

The METRO trial investigates the added value of combining MD-SBRT with time-limited intensified hormonal therapy in both synchronous and metachronous oligometastatic hormone-sensitive prostate cancer staged by PSMA‑PET/CT. The use of the PSMA-RADS scale for inclusion ensures a standardized and reproducible approach for patient selection.

**Trial registration:**

ClinicalTrials.gov Identifier: NCT04983095.

**Supplementary Information:**

The online version contains supplementary material available at 10.1186/s12885-026-15906-6.

## Background

Cumulating data from retrospective series and phase II trials suggests favourable outcome with metastasis-directed stereotactic body radiotherapy (MD-SBRT) in oligometastatic hormone sensitive prostate cancer (omHSPC) [1–3]. To optimise disease control with local treatments, such as surgery or radiotherapy, detection of all metastatic lesions is considered highly important. Prostate-specific membrane antigen-positron emission tomography/computed tomography (PSMA-PET/CT) has demonstrated superior sensitivity compared to conventional imaging both in staging of high-risk prostate cancer [4] and detecting recurrences post prostatectomy [5, 6]. Moreover, PSMA-PET/CT has shown promise in guiding MD-SBRT [2]. To date, no randomised studies on MD-SBRT including patients with de novo oligometastatic disease and time limited androgen deprivation therapy (ADT) in combination with androgen receptor pathway inhibitors (ARPi) have been published.

We present the protocol of a pragmatic trial investigating MD-SBRT alongside time-limited ADT/ARPi in patients with PSMA-PET/CT-detected omHSPC.

## Methods and design

METRO is run as an open-label, two-arm, phase III, multicentre trial. The primary objective is to evaluate whether the addition of MD-SBRT to standard of care (SoC) improves progression free survival in patients with omHSPC.

Ethical approved was granted by the Swedish Ethical Review Authority (ref. no. 2021–02766) and the Regional Committee for Medical and Health Research Ethics, Mid-Norway (ref. no. 752599) and the trial was registered 2021–07–21 at ClinicalTrials.gov (Identifier: NCT 04983095). Written informed consent is obtained by an investigator after the patient has had the opportunity to ask questions, and a signed copy is provided for the patient’s records.

**Fig. 1:**
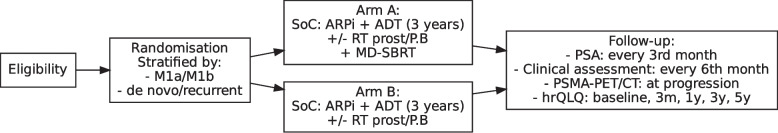
Study design for the METRO trial. M according to the TNM staging system 8^th^ edition provided by the Union for International Cancer Control (UICC)

The patients are then randomised in a 1:1 ratio to either arm A – MD‑SBRT in addition to SoC, or arm B – SoC alone (Fig. 1). Randomisation is performed within the electronic case report form (eCRF), and the randomisation sequence was generated by an independent statistician using stratified block permutation based on disease type (metachronous vs. synchronous) and metastatic site (bone vs. non‑bone). None of the study personnel have access to the randomisation sequence.Accrual is expected to conclude in early 2027, and the study is projected to complete in 2031.

### Organizational structure of the trial

The trial is an academic, investigator‑initiated study with the Cancer Center, Region Västerbotten, as the formal sponsor and coordinating site. The coordinating principal investigator, holds overall responsibility for organizing a minimum of two steering committee meetings per year, managing submissions of protocol amendments or complementary documentation to ethical review boards, overseeing funding matters, and ensuring protocol adherence including training of site personnel. The coordinating investigator also leads the integration and oversight of specialist working groups, such as those responsible for biomarker analyses or quality assurance activities.

The steering committee consists of the principal investigators from each participating site, complemented by adjunct members with defined domain‑specific responsibilities. The committee meets twice per academic term to review overarching protocol issues, discuss study progress, and evaluate all reported serious adverse events (SAEs) and suspected endpoints to ensure consistent assessment across sites.

Data management for the trial is provided by the Clinical Research Center (CRC) in Umeå, which is responsible for maintaining the study database, monitoring data quality, managing query resolution, and ensuring secure and compliant handling of participant‑level data. Additional operational groups may be engaged as needed, including those responsible for endpoint evaluation, imaging review, or other trial‑specific oversight tasks.

### Study sites

Umeå University Hospital, Umeå, Sweden.

Karolinska University Hospital, Stockholm, Sweden.

Capio St Görans Hospital, Stockholm, Sweden.

South Hospital, Stockholm, Sweden.

Örebro University Hospital, Örebro, Sweden.

Ryhovs County Hospital, Jönköping, Sweden.

Skåne University Hospital, Lund, Sweden.

Ålesund Hospital, Ålesund, Norway.

St Olavs University Hospital, Trondheim, Norway.

Benchmarking performed, not yet recruiting:

Sahlgrenska University Hospital, Gothenburg, Sweden.

### Objectives

#### Primary endpoint

- Biochemical progression free survival (bPFS): time from randomisation to biochemical progression according to EAU-ESTRO guidelines [7]:
  - ◦ In patients with de novo disease and in patients with a recurrence post-definitive radiotherapy: a rise in serum prostate-specific antigen (PSA) > 2 ng/mL above the nadir.
  - ◦ In patients with a recurrence post-prostatectomy: a rise in PSA > 0.2 ng/mL above the nadir.

#### Secondary endpoints

- Time to castration resistant prostate cancer (tCRPC): time from randomisation to biochemical progression [7] with castrate levels of testosterone (< 50 ng/dL).
- Time to next systemic therapy (tNST): time from end of systemic treatment as part of SoC to next line of systemic treatment.
- Radiological distant progression free survival (rPFS): the time from randomisation to the first documented radiological non-pelvic progression, assessed by PSMA-PET/CT using visual RECIP 1.0 [8].
- Prostate cancer specific survival (PCSS): time from randomisation to death due to prostate cancer
- Overall survival: time from randomisation to death due to any cause
- Patterns of progression
- Health related quality of life (hrQoL) assessed using the EORTC QLQ-C30 v3.0 at 3 months, and at 1, 3, and 5 years after randomisation.
- Side effects of MD-SBRT assessed using CTCAE v. 5.0 collected from randomisation to 6 months of follow-up (early) and from 6 months after randomisation to until five years of follow-up (late).
- Difference in outcome according to strata

### Explorative Biomarker Investigations

- After an additional informed consent, blood samples will be collected for tumour-derived biomarker analysis at baseline, at 1 and 3 months, and at the time of disease progression. These samples will be analysed to identify potential prognostic and predictive biomarkers that may inform individualised treatment strategies. Depending on technological advancements during the trial period, specific biomarkers such as platelets, immune profiles, cell-free DNA, and cell-free RNA will be evaluated.
- Imaging biomarkers from the diagnostic PSMA-PET/CT acquisition in all patients will be assessed for association with survival outcomes. A repeat PSMA-PET/CT will be acquired at disease progression to determine patterns of progression and to guide further treatment decisions. Both 68 Ga and 18 F are accepted PET radionuclides for labelling of PSMA, but the repeat PSMA-PET/CT must be performed using the same tracer as the initial diagnostic scan.

### Inclusion criteria

1. Histologically confirmed prostate cancer (ICD-O-3 C61)
2. World health organization (WHO) performance status 0–1
3. 1–3 skeletal or extra pelvic lymph node metastases detected by PSMA-PET/CT

### Exclusion criteria

1. Castration resistant prostate cancer (progression with castrate levels of testosterone)
2. Any treatment known to affect PSA within 6 months (exception: ADT started due to oligometastatic disease within approx. 2 weeks of study entry with collected pre-ADT PSA and testosterone)
3. Patient suitable for other treatment than standard treatment described in the protocol as judged by treating physician
4. Life expectancy < 3 years by any reason, including concomitant or previous malignancies
5. Previous radiotherapy or surgery that may interfere with the planned treatment (including intra-prostatic recurrence if previous RT to the prostate)
6. > 3 PSMA-PET/CT positive target lesions (excluding the prostate and regional lymph node metastases in in de novo patients or prostate bed and regional lymph node metastases in recurrent patients)
7. PSMA-PET/CT verified metastases other than skeletal or lymph nodes
8. Significant overlap of intended SBRT with previous RT fields or exceeded dose constraint to OAR(s) as specified in the present study protocol
9. Metastases in base of scull and/or calotte.

### Study procedures

Screening/baseline data are collected within −1/+ 5 days of randomisation, including a PSMA-PET/CT examination within 30 days of randomisation. To facilitate structured interpretation, the Prostate-Specific Membrane Antigen Reporting and Data System (PSMA-RADS) scale is used as support at patient inclusion [9, 10]. Lesions highly suspected of metastatic disease (PSMA-RADS 4–5) are considered PSMA-PET/CT positive in the present study. The study-intervention is to be initiated within a month of randomisation. The patients are then followed with visits at one and three and six months after randomisation, and thereafter every sixth month for five years. PSA is collected every three months until CRPC. If the patient is lost to follow-up, they will be followed for survival endpoints through the national quality registries of the participating countries. Additional clinical visits are scheduled upon suspected progression. An overview of the study procedures is provided in Table 1.

**Table 1:**
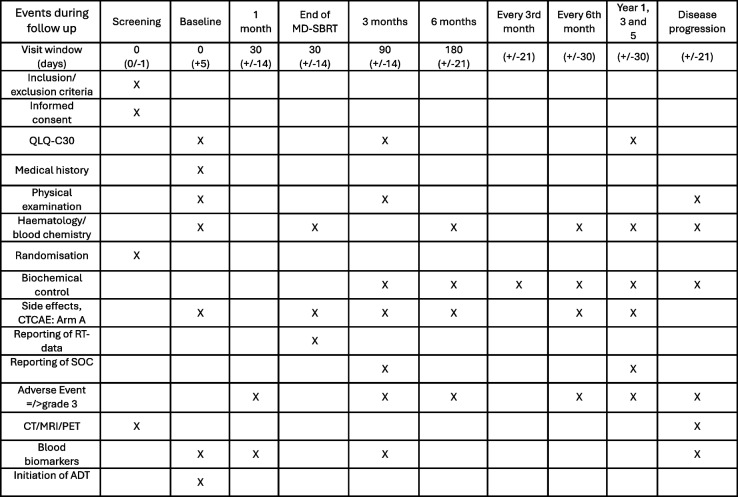
Overview of study procedures

The time points for the events during follow up are calculated from randomisation. For patients in treatment arm A) SoC + MD-SBRT the 1 month visit and the End of MD-SBRT visit can be performed at the same time

All patients in the trial are insured and eligible for compensation under the Swedish Patient Insurance/Norwegian Patient Injury Compensation.

Ancillary care, including visits required to monitor SoC, is provided as clinically indicated. Additional anti‑cancer treatments beyond SoC, such as chemotherapy, poly(ADP‑ribose) polymerase (PARP) inhibitors, or palliative radiotherapy to bone metastases, are not permitted prior to biochemical progression. Post‑trial medical care is covered by the national social insurance systems in each participating country.

### Interventions

The intention of the trial is to deliver definitive RT to all PSMA-PET/CT-positive lesions locally/regionally in both treatment arms. RT to the prostate bed in a recurrent patient without suspicion of local recurrence is however not mandatory but is decided by the investigator. SoC must be determined for each patient prior to randomisation. Detailed information on RT-set up and delivery are provided in the study protocol (available as supplementary material).

### Definition of SoC

#### Anti- hormonal treatment

ADT administered as three years of GnRH-antagonist and ARPi (1st choice: abiraterone + prednisolone) for two years following the recommendations from the STAMPEDE meta-analysis [11]. After end of ARPi, de-escalation to antiandrogen (bicalutamide) is allowed, but must be noted within the eCRF.

#### Radiotherapy

Definitive RT to the prostate will be delivered as peer investigators choice. Elective nodal irradiation is allowed. For metachronous patients post prostatectomy without a PSMA-PET-positive finding in the prostate bed, salvage RT is not mandatory and is preferably omitted for low-risk patients according to the EAU guidelines [12].

PSMA-PET/CT-positive regional lymph nodes should preferably be treated with pelvic fields in conjunction with RT to the prostate/prostate bed. SBRT to regional lymph nodes instead of pelvic fields can be used at the investigator’s discretion, mainly to avoid overlapping high dose areas with the MD-SBRT delivered to pelvic bones.

### Experimental treatment

#### Dose prescription

Metastasis-directed radiotherapy is delivered using stereotactic principles and prescribed as either 30 Gy in 3 fractions or 40 Gy in 5 fractions. An inhomogeneous dose prescription with 80% of the central dose covering the planning target volume and a central dose of a minimum of 120% of the prescribed dose is recommended. As an exception, pelvic bone metastases can be treated with conventional radiotherapy in conjunction with radiotherapy to the prostate/prostate bed to avoid overlap of treatment fields (using doses of ≥ 55 Gy in 20 fractions, ≥ 70 Gy in 35 fractions).

#### Target volumes

The clinical target volumes (CTVs) for bone lesions are defined according to international guidelines [11, 13, 14]. Spine metastases rendering a gross tumor volume (GTV) of less than 2 cm3 could be treated as a non-spine bone metastasis, at the discretion of treating physiscian. For lymph node metastases, the entire lymph node without additional margin is considered as CTV. If extra-nodal extension is suspected, a 5 mm margin is recommended.

### Radiotherapy Quality Assurance (QA)

All sites that deliver the trial intervention, MD-SBRT, have completed a site and study-specific QA-questionnaire regarding setup methods and accuracy. Moreover, a two-step benchmarking was performed before the sites were open for inclusion: two cases involving lymph node, spine and none-spinal bony metastases were distributed for target definition, centrally reviewed, and presented to each site. As a second step, treatment planning based on reference delineations was performed by all sites and centrally reviewed and presented to each site.

## Statistical considerations

### Sample size

The trial was initiated at a single site in October 2021. Lifelong ADT was considered SOC for all study participants. Based on previous trials of MD-SBRT a prolonged bPFS with a hazard ratio (HR) of 0.5 was expected for the experimental arm [1, 2]. A 2:1 randomisation (intervention:control) was planned and the sample size was set at 114 patients.

In 2023, the trial underwent major amendments (amendment application, Swedish Ethical Review Authority, ref. no. 2023–01224-0, 2023–04372-02). SOC for all participants was modified to time-limited ARPi and ADT based on the results of the STAMPEDE meta-analysis of adding ARPi to ADT and RT in high-risk prostate cancer [15]. A new 1:1 randomisation list was generated based on an anticipated median bPFS of 72 months in the standard arm [15]. No change was made to the assumed benefit of the study intervention rendering an adjusted sample size of 118 patients. The revised sample size was calculated assuming 80% power and a one‑sided type I error rate of 5%. xThe study was subsequently opened at multiple sites in Sweden and Norway.

Patients included prior to 2023 who wished to remain in the study signed the updated informed consent stating the risks and benefits of time limited ADT and their ADT was subsequently limited to three years.

### Data analysis

Kaplan–Meier curves will be used to visualize differences between treatment arms. For time‑to‑event endpoints (e.g., bPFS), participants without an event at analysis will be censored at the date of last adequate assessment. Survival endpoints (such as bPFS, rPFS, tNST and tCRPC) will be analysed using a Cox proportional hazards model. If non‑proportionality is detected, stratification variables will be included as strata. Sensitivity analyses adjusting for baseline imbalances will be performed. Missing data will be handled according to standard censoring rules for time‑to‑event outcomes, with additional sensitivity analyses conducted if patterns of missingness suggest potential bias. For the primary endpoint (bPFS), a treatment‑policy strategy will be applied: participants will remain in the risk set regardless of intercurrent events such as treatment discontinuation, or changes in background standard of care, and progression events will be defined according to the defined criteria. Patients enrolled prior to 2023 will be included in the intention‑to‑treat population, and a separate sensitivity analysis will be performed to assess the robustness of the primary results in light of the 2023 protocol amendments. Both an intention‑to‑treat (ITT) and a per‑protocol (PP) analysis will be performed. The PP population will include participants who adhered to the allocated intervention without major protocol deviations and had sufficient data for evaluation of the primary endpoint.

An interim analysis of acute toxicity will be conducted after a median follow-up of six months. No pre‑defined stopping rules or independent data monitoring committee have been implemented, as the intervention is well established and not associated with excessive toxicity [16].

### Data management

Baseline and outcome data, including patient reported hrQoL are collected at prespecified time points and stored in an eCRF developed and managed using REDCap® (Research Electronic Data Capture) provided by Umeå University, Sweden. All participant data will be handled with strict confidentiality. Authorized personnel beyond the treating physician may access medical records for clinical trial purposes. Side effects from the study intervention are reported and graded according to CTCAE v.5.0 with all serious adverse events reported within 24 h to the data centre. An independent Data Monitoring Centre at the Clinical Research Centre (Kliniskt forskningscentrum), Region Västerbotten, is responsible for the operational management of the eCRF and for conducting study monitoring in accordance with the study’s monitoring plan. This includes central data review, verification of source data, assessment of protocol adherence, and quality assurance measures performed by qualified monitors who operate independently of the study investigators. Data accuracy and integrity are ensured through central monitoring and compliance oversight. Any deficiencies in protocol adherence or data documentation identified during central monitoring will trigger expanded monitoring measures in accordance with the risk‑based monitoring plan. All imaging and MD-SBRT data are stored and managed using a study-specific, cloud-based platform compliant data protection regulations, including GDPR, with automated QA-checks, provided by Collective Minds Radiology (CMRAD®). No individual case review is planned.

## Discussion

We present the study protocol of a multicentre, prospective, randomised trial investigating the added value of MD-SBRT to intensified hormonal therapy in both synchronous and metachronous omHSPC, staged by PSMA-PET/CT. The intention of the study is to expand definitive treatment of prostate cancer, by combining ablative RT and time-limited anti-hormonal therapy.

To date, no conclusive evidence supports the use of MD-SBRT in the setting of distant omHSPC. A recent meta-analysis identified only two published randomised prospective trials in recurrent disease (metachronous), both conducted without the addition of ADT, and no randomised trials investigating MD-SBRT in de novo (synchronous) prostate cancer [16]. Nevertheless, an improved progression-free survival (PFS), with a pooled hazard ratio (HR) of 0.31 for MD-SBRT versus observation in oligometastatic PC was reported [15], enhancing the potential of the treatment strategy.

The combination of radiotherapy and time-limited hormonal therapy is well established in PC [17]. The STAMPEDE consortium demonstrated the benefit of adding ARPi to ADT and RT in high-risk PC, establishing two years of ARPi plus ADT as the current standard of care in these patients [15]. Patients with regional lymph node metastases staged by conventional imaging were included in the STAMPEDE meta-analysis, and it is reasonable to assume that our cohort of de novo patients with 1–3 metastases detected by PSMA-PET/CT largely overlaps with theirs.

For recurrent omHSPC, the standard for hormonal treatment combined with RT is less established. Six months of ADT monotherapy is commonly used in pelvic nodal oligo-recurrence [18–20]. However, in cases with distal nodal (M1a) or bone metastases (M1b), even when limited (< 4 metastases), lifelong ADT combined with ARPi is considered standard [21–23]. Since all patients in METRO are classified as M1 by PSMA-PET/CT, our recurrent cohort is not directly comparable. Emerging data from the EMBARK trial which included patients with high-risk biochemical recurrence (M0 by conventional imaging) support time-limited hormonal therapy with ADT and ARPi (enzalutamide) in a population that largely overlaps with the metachronous population in METRO [23]. Thus, time-limited intensified hormonal treatment is supported in both patient settings.

In line with this, other contemporary trials have adopted similar eligibility criteria and treatment strategies. The Oligo-PRESTO (NTC04115007) trial investigating the addition of MD-SBRT also choose to include both synchronous/metachronous omHSPC in the trial and amended their protocol to allow for intermittent hormonal treatment in 2023, similar to the METRO-trial. The first results from that trial are expected in late 2026.

Local RT to the prostate in patients with low volume metastatic disease has demonstrated an improved PFS [24]. In METRO, definitive RT-doses to the prostate are recommended, higher doses than traditionally delivered to the prostate in the metastatic setting. This could be interpreted as an overtreatment of the prostate. However, the true implication of PSMA-PET/CT detected oligo-metastases on overall survival is not yet fully established.

The role of local treatment differs substantially in the post‑prostatectomy setting. In patients with recurrence post prostatectomy without a PSMA-PET/CT detected local recurrence, the addition of salvage RT to the prostate bed is not mandatory, as the benefits of treating the prostate bed in this setting is less established.

In the changes made to the trial in 2023, we proposed that regional lymph node metastases were to be treated simultaneously with elective pelvic fields in conjunction with the treatment to the prostate/prostate bed. The recommendations are in line with the results from STORM/PEACE V-trial that demonstrated fewer recurrences with this approach than with MD-SBRT to pelvic nodal metastases [25].

The METRO trial has used the threshold of ≤ 3 metastases which is in the lower range of what’s suggested in the literature to [26]. It is however in line with previously published landmark trials in oligometastatic PC, the STOMP and ORIOLE trials [1, 2].

Biochemical PFS was selected as primary endpoint due to the well-documented long natural history of PC. Choosing an intermediate endpoint as primary is acknowledged as a major limitation of the study. However, choosing a more robust endpoint, such as radiological PFS or time to CRPC, would likely delay the reporting of results to the point where they may risk becoming outdated [27]. Nevertheless, patient follow-up will continue until progression at castrate levels of serum testosterone. At each biochemical progression, a PSMA-PET/CT will be performed to document disease status, enabling reporting of time to radiological progression.

The small sample size is recognised as another limitation. Although the power calculation is based on previous phase II trials, there is a risk that the addition of intensified hormonal therapy may dilute the observed treatment effect, reducing the magnitude of the PFS risk reduction reported in prior ADT‑free trials [1, 2].

To maximise the broader impact of the trial, detailed results will be contributed to a planned prospective meta-analysis of the effects of MD-SABR. Conducted under the auspices of the STOPCAP collaboration, this meta-analysis will enable timely, reliable and detailed evaluation of effects based on all relevant trials.

## Supplementary Information

Supplementary Material 1.

Supplementary Material 2.

## Data Availability

The datasets generated and/or analyzed during the current study are not publicly available due to the inclusion of sensitive personal data and in compliance with the General Data Protection Regulation (GDPR). However, anonymized data may be available from the corresponding author upon reasonable request and subject to applicable data protection requirements.

## Abbreviations

ADT: Androgen deprivation therapy
ARPi: Androgen receptor pathway inhibitor
bPFS: Biochemical progression free survival
CRPC: Castration-resistant prostate cancer
CT: Computed tomography
CTC: Circulating tumour cell
CTCAE: Common terminology criteria for adverse events
CTV: Clinical tumour volume
eCRF: Electronic case report form
EAU: European Association of Urology
EORTC: European Organisation for Research and Treatment of Cancer
ESTRO: European Society for Radiotherapy & Oncology
Gy: Gray
hrQoL: Health related Quality of Life
HSPC: Hormone Sensitive Prostate Cancer
ICRU: International Commission on Radiation Units and Measurements
IGRT: Image guided radiotherapy
MRI: Magnetic resonance imaging
MD-SBRT: Metastasis directed stereotactic body radiotherapy
OS: Overall survival
omHSPC: Oligometastatic Hormone Sensitive Prostate Cancer
PARP: Poly(ADP‑ribose) polymerase
PET: Positron emission tomography
PRO: Patient reported outcome
PSA: Prostate-specific antigen
PSMA: Prostate-specific membrane antigen
P:B: Prostate Bed
QA: Quality assurance
QoL: Quality of life
QLQ: Quality of life questionnaire
RT: Radiotherapy
rPFS: Radiological progression free survival
SBRT: Stereotactic body radiotherapy (extra cranial)
TNM: Tumour Node Metastasis classification
UICC: Union for International Cancer Control
VMAT: Volumetric modulated arc therapy
WHO: World Health Organization
