# Supplementary material for: Metastasis-directed SBRT for oligometastatic hormone sensitive prostate cancer (METRO): protocol for a prospective randomised phase III trial, NCT04983095
Source: BMC Cancer. 2026 Mar 25;26:456. doi: 10.1186/s12885-026-15906-6 (PMC13063559; doi:10.1186/s12885-026-15906-6)
Supplement: Supplementary file 2 — Supplementary Material 2. [file 12885_2026_15906_MOESM2_ESM.pdf]

Metastasis directed Stereotactic Body Radiotherapy for Oligo metastatic Hormone sensitive Prostate Cancer

METRO

**PI**	Karin Söderkvist
**Sponsor**	Cancer Centre<br>Umeå University Hospital<br>Region Västerbotten<br>SE 901 85 Umeå<br>Sweden
**Protocol date**	2026-01-21

Protocol Signature Sheet

Signature principal investigator

On behalf of the study group

________________________ ___________________

Karin Söderkvist
Cancercentrum
Norrlands universitetssjukhus
901 85 Umeå

Date

Contacts

**Principal investigator**	Karin Söderkvist, Oncologist<br>Norrlands universitetssjukhus<br>903 37 Umeå<br>karin.soderkvist@umu.se
**Study Group**	Mattias Hedman, Oncologist<br>Karolinska Universitetssjukhuset<br>mattias.hedman@regionstockholm.se<br><br>Chunde Li, Oncologist<br>Södersjukhuset, Stockholm<br>chunde.li@regionstockholm.se<br><br>Martina Westin<br>Capio S:t Görans sjukhus<br>martina.westin@capiostgoran.se<br><br>Kirsten Björnlinger, Oncologist<br>Länssjukhuset Ryhov, Jönköping<br>kirsten.bjornlinger@rjl.se<br><br>Jon Kindblom, Oncologist<br>Sahlgrenska universitetssjukhuset, Göteborg<br>jon.kindblom@vgregion.se<br><br>Jenny Kahlmeter-Brandell, Oncologist<br>Örebro Universitetssjukhus, Örebro<br>jenny.kahlmeter-brandell@regionorebrolan.se<br><br>Adalsteinn Gunnlaugsson, Oncologist<br>Skånes Universitetssjukhus, Lund<br>adalsteinn.gunnlaugssson@skane.se<br><br>Bjørg Yksnøy Aksnessæther, Oncologist<br>Ålesund Sjukehus<br>bjorg.y.aksnessether@helse-mr.no<br><br>Natalie Skorve, Oncologist<br>St. Olavs Hospital, Trondheim<br>natalie.skorve@stolav.no
**Imaging biomarkers**	Katrine Riklund, Prof.<br>Dep of Diagnostics & Intervention, Umeå University<br>Sara Strandberg, Associate prof.<br>Dep of Diagnostics & Intervention, Umeå University

Camilla Thellenberg-Karlsson, Associate prof.
Dep of Diagnostics & Intervention, Umeå University

Blood biomarkers

Pernilla Wikström, Prof.
Dep of Medical Biosciences, Umeå University

RT QA Group

Joakim Jonsson, radiation physicist
Norrlands universitetssjukhus, Umeå
joakim.jonsson@regionvasterbotten.se

Kristin Karlsson, radiation physicist
Karolinska Universitetssjukhuset, Stockholm
kristin.a.karlsson@regionstockholm.se

Essie Persson, radiation physicist
Örebro Universitetssjukhus, Örebro
essie.persson@regionorebrolan.se

Emma Djärv, radiation physicist
Sahlgrenska universitetssjukhuset, Göteborg
emma.djarf@vgregion.se

Jakob Eriksson, radiation physicist
Länssjukhuset Ryhov, Jönköping
jakob.eriksson@rjl.se

Jonas Scherman, radiation physicist
Skånes Universitetssjukhus, Lund
jonas.scherman@skane.se

Marie Johansen, radiation physicist
marie.johansen@helse-mr.no

Kajsa Fridström, radiation physicist
St. Olavs Hospital, Trondheim
kajsa.fridstrom@stolav.no

**Data center and monitoring**	Clinical Research Center<br>Umeå University Hospital<br>kliniskforskningscentrum@regionvasterbotten.se<br>Contacts:<br>Ida Lundberg, project manager<br>ida.v.lundberg@regionvasterbotten.se
**Statistician**	Ove Björ, statistician<br>RCC Norr, Umeå<br>ove.bjor@regionvasterbotten.se<br>Björn Tavelin, statistician<br>RCC Norr, Umeå<br>bjorn.tavelin@umu.se
**Steering committee**	The steering committee comprises the study group, representatives from the QA and biomarker groups, and study statisticians.

1 Synopsis

**Protocol title**	Metastasis directed stereotactic body radiotherapy for oligometastatic hormone sensitive prostate cancer, METRO
**NCT number**	**NCT04983095**
**Development phase**	Phase III
**Study population**	Patients with hormone sensitive prostate cancer (HSPC) with oligometastatic disease detected by PSMA-PET/DT. This includes patients with de novo oligometastatic HSPC and recurrent HSPC after primary RT or prostatectomy.
**Endpoints**	**Primary endpoint**<br>• Biochemical progression free survival (bPFS)<br>**Secondary endpoints**<br>• Acute side effects after MD-SBRT (CTCAE ver.5.0)<br>• Late side effects after MD-SBRT (CTCAE ver.5.0)<br>• QoL at 3 months, 1, 3 and 5 years (EORTC QLQ-C30 v3.0)<br>• Time to castration resistant prostate cancer (tCRPC)<br>• Radiological distant progression free survival (rPFS)<br>• Prostate cancer specific survival (PCSS)<br>• Overall survival (OS)<br>• Patterns of progression<br>**Explorative biomarker investigations**<br>• Predictive value of novel biomarkers in blood, tissue and imaging
**Study design**	The study is an open label, multi-centre, permuted block randomisation study. The patients will be randomised in a 1:1 ratio to treatment consisting of<br>• Arm A: MD-SBRT in addition to standard treatment<br>• Arm B: Standard treatment
**Eligibility criteria**	**Inclusion criteria**<br>1. Histologically confirmed prostate cancer (ICD-O-3 C61)<br>2. WHO/ECOG performance status 0-1<br>3. 1-3[1] skeletal or extra pelvic lymph node metastases detected by PSMA-PET/CT[2] in<br>i. de novo prostate cancer or<br>ii. PSA-relapse after definitive RT or prostatectomy<br>4. Willing and able to provide informed consent

[1] *Multiple lymph nodes in close vicinity (<1 cm) may be counted as one target lesion if they render one planning target volume (PTV) according to section 12.2.1*

[2] *As judged per PSMA-RADS, see AD III*

Exclusion criteria

1. Castration resistant prostate cancer (progression with castrate levels of testosterone)
2. Any treatment known to affect PSA, within 6 months (exception: ADT started due to oligometastatic disease within 2 weeks of randomisation with collected pre-ADT PSA and testosterone pre-ADT)
3. Patient suitable for other treatment than standard treatment described in the protocol as judged by treating physician
4. Life expectancy <3 years by any reason, including concomitant or previous malignancies
5. Previous radiotherapy or surgery that may interfere with the planned treatment (including intra-prostatic recurrence if previous RT to the prostate)
6. > 3 PSMA-PET/CT positive target lesions (excluding the prostate and regional lymph node metastases in in de novo patients or prostate bed and regional lymph node metastases in recurrent patients)
7. PSMA-PET verified metastases other than skeletal or lymph nodes
8. Significant overlap of intended SBRT with previous RT fields or exceeded dose constraint to OAR(s) as specified in study protocol
9. Metastases in base of scull and/or calotte

Stratification

The randomisation will be stratified to balance treatment arms for de novo oligo-metastatic and oligo-recurrent patients as well as for patients with lymph node metastases only and patients with bone metastases and/or lymph node metastases.

Standard of Care

Arm A and B: ADT is administered from the time of informed consent. The ADT is restricted to 3 years. 2nd generation hormonal therapy (ARPI) with 2 years Abiraterone acetate + prednisolone is included in the standard treatment for all patients.
If the patient is de novo oligo-metastatic, RT to the prostate +/- pelvic fields is considered as standard treatment. In patients with relapse, salvage RT to the prostate bed +/- pelvic fields is optional if there is no locoregional relapse detected by PSMA-PET/CT.

Study intervention

Arm A: MD-SBRT to all PSMA-PET/CT positive metastatic target volume(s) with 30 Gy in 3 fractions or 40 Gy delivered in 5 fractions in addition to standard treatment. For patients with a recurrence post prostatectomy and a PSMA-PET-positive finding in the prostate bed, salvage RT to the prostate bed is mandatory. PSMA-PET-positive regional lymph nodes are to be treated for all patients in the intervention arm, preferably with pelvic fields.

**Efficacy control**	Biochemical progression free survival<br>Time to CRPC<br>Radiological progression free survival<br>Overall survival
**Safety evaluation**	Acute and late side effects; Adverse events and side effects graded according to CTCAE v5.0. Serious Adverse Events are to be reported within 24 hours throughout the study duration.
**Quality of Life**	Patient scored morbidity and health related quality of life (hrQoL) assessed by EORTC QLQ30
**Statistical methods**	**Efficacy**: Randomisation time is set as baseline time. Primary endpoint will be analysed using a Cox proportional hazards model. If non-proportionality is detected, stratification variables will be included as strata. Sensitivity analyses adjusting for baseline imbalances will be performed, and Kaplan–Meier curves will be used to visualize treatment groups.<br>**Safety**: Mann-Whitney U-test or Fishers exact test
**Criteria for evaluation**	**Per protocol**: Patients treated according to protocol<br>**Intention to treat**: All included patients
**Planned sample size**	118 patients
**Screening procedure**	Inclusion/exclusion criteria evaluation including evaluation of feasibility of RT to all positive lesions on PSMA-PET/CT performed within 30 days of randomisation.
**Study specific procedure**	Collecting/Recording of baseline data including baseline PROM.<br>Standard of care include ADT initiated at randomisation and abiraterone initiated within 8 weeks of randomisation to all study participants.<br>Start of study intervention with MD-SBRT within 28 days of randomisation to patients in arm A.<br>RT to prostate/prostate bed +/-pelvic lymph nodes started within »90 days as part of standard of care.<br>Follow up according to protocol.
**End of study**	Minimum 5 year follow up.
**Analysis plan**	The primary end point will be analysed after pre-specified number of events have occurred. Safety analysis of acute side effects will take place after median follow up of 6 months. Safety analysis of late side effects will be analysed after median follow up of 60 months.
**Duration of the study**	Three to five years inclusion. 72 months of follow-up after randomisation of the last patient is expected.

ADT	Androgen deprivation therapy
AD	Addendum
AE	Adverse event
AJCC	American Joint Committee on Cancer
ARPi	Androgen receptor pathway inhibitor
bPFS	Biochemical progression free survival
CRF	Case report form
CRPC	Castration-resistant prostate cancer
CT	Computed tomography
CTC/CTDNA	Circulating tumour cell/circulating tumour DNA
CTCAE	Common terminology criteria for adverse events
CTV	Clinical tumour volume
eCRF	Electronic case report form
EORTC	European Organisation for Research and Treatment of Cancer
FFS	Failure free survival
FTV	Functional tumour volume
GTV	Gross tumour volume
Gy	Gray
hrQoL	Health related Quality of Life
HSPC	Hormone Sensitive Prostate Cancer
IC	Informed Consent
ICRU	International Commission on Radiation Units and Measurements
IGRT	Image guided radiotherapy
MRI	Magnetic resonance imaging
MD-SBRT	Metastasis directed stereotactic body radiotherapy
MV	Mega volt
OMPC	Oligo-metastatic prostate cancer
OS	Overall survival
PCSS	Prostate cancer specific survival
PET	Positron emission tomography
PRO	Patient reported outcome
PRV	Planning organ at risk volume
PSA	Prostate-specific antigen
PSMA	Prostate-specific membrane antigen
PTV	Planning target volume
QA	Quality assurance
QoL	Quality of life
QLQ	Quality of life questionnaire
ROI	Region of interest
RT	Radiotherapy
RTOG	Radiation Therapy Oncology Group
rPFS	Radiological distant progression free survival
SAE	Serious adverse event
SBRT	Stereotactic body radiotherapy (extra cranial)
TLG	Total lesion glycolysis
TNM	Tumour Node Metastasis classification
VMAT	Volumetric modulated arc therapy

WHO/ECOG	World Health Organization/Eastern Cooperative Oncology Group

2 Table of contents

1 Synopsis **6**

2 Table of contents **11**

3 Background and introduction **14**

3.1 Study rationale *14*

3.2 Background *14*

4 Objectives of the trial **15**

4.1 Primary objective *15*

4.2 Secondary objectives *15*

5 End points **15**

5.1 Primary end point *15*

5.2 Secondary end points *16*

6 Study design **16**

7 Study period **16**

8 Overview of study treatment **16**

9 Patient selection criteria **17**

9.1 Inclusion criteria *17*

9.2 Exclusion criteria *17*

10 Study procedures **18**

10.1 Screening procedure *18*

10.1.1 Informed consent 18

10.1.2 Screening evaluations 18

10.2 Baseline evaluations *18*

10.3 1 month after randomisation *18*

10.4 End of MD-SBRT *19*

10.5 Evaluation 3 months after randomisation *19*

10.6 Evaluation 6 months after randomisation *19*

10.7 Every 3rd month until end of study *19*

10.8 Every 6th month until 60 months *19*

10.9 Year 1, 2, 3 and 5 .... *19*

10.10 Disease progression *19*

10.11 End of study *20*

11 Standard of care (Arm A and B) **20**

11.1 Androgen deprivation therapy and androgen receptor pathway inhibitor *20*

11.2 Radiotherapy ... *20*
11.2.1 Radiotherapy in de novo patients ... 20
11.2.2 Radiotherapy in patients with relapse ... 21

12 Stereotactic body radiotherapy ... 21

12.1 Patient position and immobilisation ... *21*

12.2 Patient data acquisition ... *22*

12.3 Motion assessment ... *22*
12.3.1 Periodic movements ... 22
12.3.2 Non-periodic movements ... 23

12.4 Volume delineation ... *23*
12.4.1 Target volumes ... 23
12.4.2 Organ(s) at risk ... 24

12.5 Structure names in the treatment planning system ... *25*

12.6 Treatment planning ... *25*
12.6.1 Beam characteristics ... 25
12.6.2 Dose-volume objectives ... 25
12.6.3 Dose-volume constraints ... 26
12.6.4 Dose calculation ... 26

12.7 Fractionation (scheduling) ... *27*

12.8 Online isocenter localisation ... *27*

12.9 Adaptive planning ... *27*

12.10 SBRT treatment details ... *27*
Table 1. Overview of fractionation schedules and target volumes by location ... 28
12.10.1 Treatment details lymph nodes ... 28
12.10.2 Treatment details spinal lesions ... 29
12.10.3 Treatment details non-spinal bony lesions ... 30

12.11 Radiotherapy, quality assurance (RT-QA) ... *31*

12.12 Dummy run procedure ... *31*

12.13 Individual patient checks ... *31*

13 Patient reported outcome (PRO) and health related QoL ... 31

14 Statistical plan ... 31

14.1 Sample size determination ... *31*

14.2 Statistical methods and analysis plan ... *32*

14.3 Subject populations(s) for analysis ... *32*

15 Safety reporting ... 33

15.1 Definitions of Serious Adverse Event (SAE) ... *33*

15.2 Reporting procedure ... *33*

16 Quality assurance ... 33

16.1 Control of data consistency ... *33*

16.2 Site monitoring ... *34*

16.3 Legal aspects ... *34*

16.4 Direct access to data/documents ... *34*

17 Data management ... 34

17.1 Patient registration/randomisation procedure ... *34*
17.2 Case report forms ... *34*
17.3 Health related quality of life forms - PRO ... *34*
17.4 Imaging and RT data ... *35*
18 Exploratory biomarkers ... 35
18.1 Blood biomarkers ... *35*
18.2 PSMA-PET biomarkers ... *35*
19 Ethical considerations ... 36
19.1 Patient protection ... *36*
19.2 Patient integrity ... *36*
19.3 Informed consent ... *36*
19.4 Independent ethical review ... *36*
19.5 Risk benefit analysis ... *37*
20 Participating centres ... 37
21 Ownership of data ... 37
21.1 Meetings ... *37*
21.2 Reporting ... *38*
22 Flow chart of study procedures ... 39
23 References ... 40

3 Background and introduction

3.1 Study rationale

Cumulating data from retrospective series and phase II trials suggests favourable outcome with metastasis directed radiotherapy in oligometastatic prostate cancer. This multicentre randomised phase III study aims to investigate the possible benefit of metastasis directed stereotactic body radiotherapy (MD-SBRT) in addition to standard treatment in oligo metastatic hormone sensitive prostate cancer (HSPC) staged with Prostate Specific Membrane Antigen (PSMA) Positron Emission Tomography (PET)/computerised tomography (CT).

3.2 Background

The concept of oligo metastatic disease as a separate stage in the development of generalised prostatic cancer has been presented in the early nineties. It is hypothesised that the cancer cells in oligo metastatic disease does not yet have their full potential for dissemination, and thus local treatment could prolong or even prevent further spread (1). This concept has been utilized for other diagnoses (2-5). Retrospective series have presented advantageous result of metastasis directed radiotherapy, as well as two recent randomised phase II studies (6, 7). This development has prompted a consensus document for oligometastatic disease from a radiotherapy point of view (8).

A prerequisite for disease control is that all metastatic lesions are treated. In a recently published randomised multicentre trial PSMA-PET has demonstrated superiority in staging high risk prostate cancer compared to conventional imaging with CT and bone scan (9). The PSMA-RADS version 1.0 has been proposed as a standardized system to report and interpret PSMA-PET/CT in PSMA targeted imaging system, where lesions are graded in a 5 point scale, with 4-5 as positive for metastatic disease (10, 11). The phase II study ORIOLE regarding SBRT for oligo-metastatic prostate cancer supported the hypothesis of PSMA-PET/CT directed radiotherapy (6). Patients with all PSMA-PET/CT detected lesions treated demonstrated an improved failure free survival (HR=0.26) as compared to those with untreated PSMA-positive lesions.

In the prospective randomised phase II study STOMP by Ost et. al on oligo metastatic prostate cancer the primary endpoint of hazard ratio (HR) 0.5 for androgen deprivation therapy (ADT)-free survival at 12 months was not met (7). However, a significantly improved ADT-free survival at 12 months (HR=0.6) was demonstrated. In the STOMP-trial, the beneficial effect of adding ADT to RT was not utilized, and this was discussed as one of the possible contributors of the primary endpoint not being met. Moreover, ADT is considered gold standard in established metastatic disease.

Patients diagnosed with metastatic prostate cancer de novo have been demonstrated to have inferior survival versus patients with recurrent metastatic prostate cancer (12). A possible reason could be that metastases are detected at an earlier stage after primary treatment with curative intent due to scheduled follow up. It has also been hypothesised that de novo prostate cancer is a separate biological entity with increased inherent ability for metastatic spread (12, 13). Resent development with local RT for low volume disease has improved survival for the de novo patients with metastases <5 (14). Studies on metastasis directed therapy up to date has not included de novo patients, nor is the prognosis for this de novo oligo-metastatic prostate cancer given recent treatment and diagnostic advances well described in the literature. For both de novo and recurrent metastatic HSPC the

addition of docetaxel/new generation hormonal agents to ADT has been established as gold standard, however, the benefit is most pronounced for more advanced disease. Moreover, none of the landmark trials include staging by PSMA-PET/CT implying a risk of stage migration by extrapolating these results on the present study population (15).
On the other hand the meta-analysis from the STAMPEDE platform has established the addition of 2 years of Abiraterone acetate and prednisolone (hereafter referred to as Abiraterone) to 3 years of ADT in definitive RT in de novo high-risk prostate cancer, including N+ disease, staged by standard imaging (16). It is reasonable to extrapolate these results to an oligometastatic patient cohort staged by the more sensitive method, PSMA-PET/CT. Data on time limited ADT in conjunction with RT for oligo-recurrent prostate cancer patients are also emerging (17).

4 Objectives of the trial

4.1 Primary objective

To compare the progression free survival with MD-SBRT in addition to standard treatment with standard of care (SoC) alone in patients with hormone sensitive oligometastatic prostate cancer.

4.2 Secondary objectives

To compare

- Time to castration resistant prostate cancer (tCRPC)
- Time to next systemic therapy (tNST)
- Radiological distant progression free survival (rPFS)
- Prostate cancer specific survival (PCSS)
- Overall survival (OS)
- Patterns of progression
- Healt related Qality of Life (QoL) at 1, 3 and 5 years

in

A) patients with MD-SBRT + SoC
B) patients with SoC alone.

To report

- Acute and late side effects of MD-SBRT in addition to SoC
- Blood and imaging biomarkers for outcome prediction to enable individualised selection of patients for MD-SBRT
- Difference in outcome for de novo and recurrent patients.

5 End points

5.1 Primary end point

- Biochemical progression free survival (bPFS)

Defined as time from randomisation to biochemical progression without backdating.
In patients with a remaining prostate (de novo or recurrent post RT) a biochemical progression is defined as a rise in PSA >2 µg/L above nadir followed by a sequentially equal or higher PSA, minimum 4 weeks apart (18).

In recurrent patients post prostatectomy biochemical progression is defined as a rise in PSA of 0.2 µg/L above the PSA nadir followed by a sequentially equal or higher PSA, minimum 2 weeks apart (19).

5.2 Secondary end points

- Predictive value of investigated biomarkers in blood and imaging
- Acute side effects after MD-SBRT (CTCAE v 5.0)
- Late side effects after MD-SBRT (CTCAE v 5.0)
- QoL at 3 months, 1, 3 and five years
- Time to CRPC (tCRPC)
- Radiological progression free survival (rPFS)
- Time to next systemic treatment
- Patterns of progression
- Overall survival (OS)
- Differences in outcome between patients by strata

6 Study design

The study is an open label, multi-centre, randomised phase III study. 118 patients with oligo-metastatic prostate cancer will be randomised in 1:1 ratio to

- Arm A: MD-SBRT+ SoC
- Arm B: SoC

Randomisation will be stratified by de novo vs recurrent oligo-metastatic prostate cancer as well as lymph node metastases only versus bone with/without lymph node metastases using stratified permuted block randomisation.

7 Study period

The inclusion is estimated to 3-5 years. Primary endpoint is analysed at specified no of events, minimum 72 months of follow up is expected. 60 months of follow up of toxicity in experimental arm is mandatory.

8 Overview of study treatment

All patients are treated with 3 years of ADT and 2 years of Abiraterone, initiated at the time of informed consent as part of standard of care. If the patient is de novo oligo-metastatic, radiotherapy to the prostate according to local practise at the treating centre will be delivered to all study participants as part of standard treatment, see section 11.2. For de novo patients with PSMA-PET/CT detected regional lymph node metastases (N1), these are preferably treated concurrent with pelvic fields, and only as an exception with SBRT. For patients with recurrence post prostatectomy, salvage RT to the prostate bed can be delivered at the discretion of treating physician, see section 11.3. If a recurrent patient has pelvic lymph node metastases concurrent with distant metastases, these should preferably be treated with pelvic fields, and only as an exception with SBRT. The same principles are to be applied on recurrent patients post RT randomised to arm A if possible, due to previous RT to the prostate.

In addition, patients randomised to intervention arm (A) will receive metastasis directed radiotherapy delivered with stereotactic body radiotherapy (SBRT) principles. The prescription dose to the planning target volume (PTV) is 30 Gy in 3 fractions delivered every other to every fourth day or 40 Gy delivered in 5 fractions, with 1-3 days between fractions. These treatment times refers to each isocentric treatment. If multiple targets with different isocentres, they can be treated alternating days. Hence, total treatment time is dependent on number and anatomical separation of metastasis. Treatment is prescribed to the periphery of the target, generally to the 80% isodose as a percentage of a normalised maximum dose value in the centre of the GTV (i.e., with a maximum dose of 125% of the prescribed dose to PTV), for details on fractionation and dosage, see section 12.7 and 12.10.

NOTE! **Re-irradiation is not permitted (significant overlap with previous radiation fields).**

9 Patient selection criteria

9.1 Inclusion criteria

1. Histologically confirmed prostate cancer (ICD-O-3 C61).
2. WHO/ECOG performance status 0-1.
3. 1-3[3] skeletal or extra pelvic lymph node metastases detected by PSMA-PET/CT [4] in
    i. de novo oligo-metastatic cancer.
    ii. PSA-relapse after definitive RT or prostatectomy.
4. Willing and able to provide informed consent.

9.2 Exclusion criteria

1. Castration resistant prostate cancer (progression with castrate levels of testosterone)
2. Any treatment known to affect PSA within 6 months (exception: ADT started due to oligometastatic disease within approx. 2 weeks of study entry with collected pre-ADT PSA and testosterone)
3. Patient suitable for other treatment than standard treatment described in the protocol as judged by treating physician
4. Life expectancy <3 years by any reason, including concomitant or previous malignancies
5. Previous radiotherapy or surgery that may interfere with the planned treatment (including intra-prostatic recurrence if previous RT to the prostate)
6. > 3 PSMA-PET/CT positive target lesions (excluding the prostate and regional lymph node metastases in de novo patients or prostate bed and regional lymph node metastases in recurrent patients)
7. PSMA-PET/CT verified metastases other than skeletal or lymph nodes
8. Significant overlap of intended SBRT with previous RT fields or exceeded dose constraint to OAR(s) as specified in the present study protocol
9. Metastases in base of scull and/or calotte

---

[3] *Multiple lymph nodes in close vicinity (<1 cm separation) may be counted as one target lesion if they render one planning target volume (PTV) according to 12.10.1.*
[4] *See AD III.*

10 Study procedures

Planned study visits, their acceptable visit window and study activities to be performed at the visits are described by the study flow chart, see section 22.

10.1 Screening procedure

10.1.1 Informed consent

When a subject is invited to take part in the study, the investigator must adequately explain all pertinent information about the study. Patient information includes, but is not limited to, study objectives, procedures, risks, or inconveniences involved, and the right to withdraw from the study at any time. The investigator must provide the subject with a copy of the subject information sheet and then allow the subject sufficient time to consider the proposal. The investigator must also provide the subject with an opportunity to ask any questions before deciding whether to participate or not. The subject must have all information on alternative treatments in case he does not wish to participate in the study. The investigator must obtain a subject's written informed consent before any study related activity begins, including screening assessments. Both the subject and the investigator must sign and personally date the informed consent form. The subject will receive a copy of the signed form.

10.1.2 Screening evaluations

1 Check of inclusion/exclusion criteria.
2 Evaluation of the diagnostic PSMA-PET/CT performed within approx. 30 days before screening visit to assess inclusion criteria 3 and exclusion criteria 5-8 by an investigator familiar with PSMA-RADS and the MD-SBRT intended in the present trial.
3 Document patient category, M1a or M1b-status, overall PSMA-RADS score, and number of potential MD-SBRT targets.
4 Informed consent.

10.2 Baseline evaluations

1. Medical history of prostate cancer including previous treatment for localised prostate cancer (if recurrent disease).
2. PSMA-PET/CT details.
3. Medical history, including concomitant medications and smoking status.
4. Physical examination including WHO performance status.
5. Haematology: Hb, WBC, platelets.
6. Blood chemistry: PSA and testosterone (before start of ADT).
7. Blood chemistry: Bilirubin, ALP, ALAT, ASAT, creatinine, Na, K, Ca, albumin.
8. EORTC QLQ-C30 (version 3.0).
9. If randomised to Arm A: Baseline recording of toxicity according to CTCAE v5 from relevant organ(s) at risk (OARs) that lie within 3 cm of the intended target volume for the MD-SBRT.
10. Blood sample for biomarkers if applicable.

10.3 1 month after randomisation

1. Blood sample for biomarkers if applicable.

2. Adverse event grade 3 and higher.

10.4 End of MD-SBRT

Arm A only, can be integrated with 1 month visit.

1. Recording of side effects according to CTCAE v5.0 (including blood chemistry if applicable) for all organs within.
2. Reporting of MD-SBRT data and PSMA-PET/CT according to AD II.

10.5 Evaluation 3 months after randomisation

1. Physical examination.
2. Biochemical control; PSA, testosterone.
3. Adverse event grade 3 and higher.
4. EORTC QLQ-C30 (version 3.0).
5. Arm A: Recording of side effects according to CTCAE v5.0 for OARs within 3 cm of PTV (including blood chemistry if applicable).
6. Blood sample for biomarkers if applicable.
7. Reporting of treatment details delivered as part of SoC.

10.6 Evaluation 6 months after randomisation

1. Biochemical control; PSA.
2. Adverse event grade 3 and higher.
3. Arm A: Recording of side effects according to CTCAE v5.0 for OARs within 3 cm of PTV (including blood chemistry if applicable).

10.7 Every 3rd month until end of study

1. Biochemical control; PSA (ND in CRPC).

10.8 Every 6th month until 60 months

1. Biochemical control; PSA (ND in CRPC).
2. Adverse event grade 3 and higher (ND in CRPC).
3. Arm A: Recording of side effects according to CTCAE v5.0 (including blood chemistry if applicable).

10.9 Year 1, 2, 3 and 5

1. EORTC QLQ-C30 (version 3.0) at year 1,3 and 5.
2. Reporting of treatment details delivered as part of SoC at year 1, 2, and 3, including stopping date of ADT/ARPi.

10.10 Disease progression

1. Physical examination.
2. Imaging with **PSMA-PET/CT.**

3. Specify type of progression and subsequent mode of action.
4. Blood sample for biomarkers if applicable.

10.11 End of study

Performed at:
Arm A: Disease progression CRPC and 60 months of follow up of side effects/reporting of SAE.
Arm B: Disease progression CRPC

1. Reason for EOS.
2. All required data collected and monitored.

11 Standard of care (Arm A and B)

In the present setting of early detected minimal disease and close follow up it must be deemed acceptable to abstain from early chemotherapy and/or life-long hormonal agents, otherwise the patient is not eligible for the present study (exclusion criteria no 1). The patient should not be a candidate for palliative RT due to symptomatic bone metastasis (exclusion criteria no 1).

11.1 Androgen deprivation therapy and androgen receptor pathway inhibitor

ADT in the present protocol includes medical castration with GnRH-agonist, surgical castration or GnRH-antagonist, and is to be initiated for all patients at the time of informed consent. As *an* ***exception***, ADT initiated within two weeks of study entry is allowed ***if*** baseline blood chemistry including testosterone within normal bounders and pre-treatment PSA is collected. The ADT and Abiraterone is administered in concordance with (16); 2 years of Abiraterone and Prednisolone and 3 years of ADT in both treatment arms (A and B). De-escalation to 1st generation ARPi only after 2 years is permitted but must be documented in eCRF. Switch from Abiraterone to other 2nd generation ARPi due to side effects or other medical considerations is permitted but must be documented in eCRF.

11.2 Radiotherapy

The RT should be optimised to achieve disease control but to minimise potential toxicity according to local practice. It is recognised that the present population differs from patients normally treated with definitive RT to the prostate/prostate bed. **It therefor is strongly recommended that dose constraints not be exceeded, but rather to compromise coverage of the PTV in a trade of.**

11.2.1 Radiotherapy in de novo patients

Radiotherapy to the prostate as part of standard treatment should preferably be timed with the follow up visit 3 months after study entry, to avoid that toxicity from local treatment obscure the toxicity scoring from the MD-SBRT or the PROM. The local RT to the prostate for de novo patients classified as T1-3a is to be delivered according to local practice at the participating centres. 42,7 Gy in 7 fractions over 3 weeks according to the HYPO-trial (20), moderate hypofractionation according to the CHHiP-trial (21), or conventionally fractionated

regimens including concomitant pelvic lymph node fields +/- HDR-brachytherapy according to local practice at the participating centres are suggested. Neither of these schedules have demonstrated safety in the T3b-T4 setting. Therefore, patients with T3b-4a according to UICC TNM 8 can be considered for moderate hypo-fractionation of 55 Gy in 20 fractions daily over 4 weeks according to the STAMPEDE, however, the intention of the present trial is that all disease should be feasible to treat to definitive doses (prostate ≥74 Gy, lymph node metastases ≥68 Gy, EQD2 α/β=2).
PSMA-PET/CT-positive regional lymph nodes should preferably be treated with pelvic fields in conjunction with RT to the prostate/prostate bed. SBRT to regional lymph nodes instead of pelvic fields can be used at the investigator's discretion, mainly to avoid overlapping high dose areas with the MD-SBRT delivered to pelvic bones. Dose escalation to definitive doses is mandatory to bulky lymph node metastases (short axis diameter ≥1cm).

11.2.2 Radiotherapy in patients with relapse

Arm A: If PSMA-PET detects a local recurrence in the prostate bed post prostatectomy, salvage-RT is mandatory. A simultaneously integrated boost (SIB) to the PET-positive lesion is recommended. The treatment should preferably be initiated within 30 days of the start of ADT to facilitate the identification of the GTV for the SIB in the imaging required for dose planning. IGRT with VMAT is mandatory. In short; the suggested treatment is 2 Gy daily 5 days/week to 70 Gy to the prostate bed with daily image guidance (CBCT) and a SIB of 2.2 Gy daily 5 days/week to a total dose of 77 Gy to the PSMA-PET-positive lesion. In case of regional lymph node metastases these are preferably treated with pelvic lymph node-fields, with a boost to 70 Gy in a 35 fraction schedule to bulky metastases. The GTV for the SIB should be delineated with guidance of the PSMA-PET (cognitive or co-registered) and preferably with a co-registered MRI. The CTV for prostate bed is to be contoured according to international guidelines (22-24). PTV-margins are to reflect local practice at the participating centres.

Adjuvant treatment to a PSMA-PET/CT negative prostate bed and/or pelvic nodes is allowed in the present protocol primary to patients belonging to EAU high-risk group at the discretion of the treating physician.

Arm B: Salvage RT to the prostate bed or pelvic lymph nodes is allowed at the discretion of the treating physician at each centre.

NOTE! **Re-irradiation is not permitted (overlap with previous high dose-volumes).**

12 Stereotactic body radiotherapy

12.1 Patient position and immobilisation

Treatment must be setup using reproducible positioning and verified using a daily on-line imaging protocol with cone beam computerised tomography (CT). Immobilisation is executed according to individual institutional practice when delivering SBRT. Use of a stereotactic body frame with an individualised vacuum cushion as a custom immobilisation

device is recommended. However, it is not mandatory if a high degree of accuracy for immobilisation and set-up has been demonstrated by the centre pre study entry. This means that all centres should assess and document the accuracy of their immobilisation device/s used for positioning patients for SBRT. The documents must be approved by QA-group before study entry.

12.2 Patient data acquisition

Computed tomography (CT) scans with high resolution are mandatory, with the following maximum voxel sizes:

- In plane voxel size (x, y) ≤ 1mm
- Reconstructed slice thickness voxel size (z) ≤ 2mm (spine 1mm recommended)

The extent of the CT must cover all relevant OARs (as a rule of thumb within 3 cm of PTV). If dose constraint is based on total volume of OAR (e.g., kidney or liver) the entire organ must be included. Contrast may be used according to local protocols at each participating centre.

Magnetic resonance imaging (MRI) for co-registration to improve accuracy in GTV and organ at risk delineation is recommended for all sites, and mandatory for spinal treatment. The sequences should give sufficient combined resolution to determine the tumour extent on a 1x1x1 mm scale. The following sequences are suggested:

- T1 and T2 TSE Sag
- T2 and STIR TSE Ax
- T1 (+Gd) FATSAT/DIXON 3D/Sag/Cor/Ax

The MRI should be performed in treatment position (for co-registration). This is of special importance in spinal treatment. Sub-volume image registration is mandatory and must apply to the affected vertebra in spinal treatment. Introduced uncertainties must be considered and incorporated in the margins applied to the ROIs.

12.3 Motion assessment

12.3.1 Periodic movements

For targets in the thoracic wall and adjacent to the diaphragm where breathing movements can be expected to influence the target movement, the 4-dimensional CT (4DCT) and the time-weighted average CT (TW-AvCT) has become a standard to determine the extent and the probability distribution of the target movement.

Assessment of the tumour motion amplitude in x, y and z directions will be performed according to local routine at the participating centres. For all targets affected by breathing motions the concept of internal target volume or the mid-ventilation concept (with added PTV-margin) using the 4DCT should be considered. If periodic movements in the target exceed 5 mm in any direction options for motion management for example, treatment in breath hold or abdominal compression should be considered to avoid unnecessary irradiation of surrounding tissue.

12.3.2 Non-periodic movements

This section applies to targets not expected to be influenced by breathing motion, such as spine, retroperitoneal and pelvic lymph nodes.

Repeated CT scans separated by 10 minutes to assess intra-fraction motions, e.g., peristaltic movements and baseline shifts of the target is highly recommended. The 10 min scan difference yields important information about the performance of the fixation for a specific patient. 10 min is a representative time windows for the process (in the treatment facility) of image acquisition, on-line match and irradiation. Observed base line shifts must be considered by the physician delineating the target volumes when deciding the adequacy of the planning risk volume (PRV) and a PTV(s). A CT performed at a different date must be evaluated for significant changes in the configuration of some OARs (more specifically the bowel, bladder, rectum and stomach. This can be done by a repeated dose-planning CT the following day (recommended) or by evaluation of the diagnostic PSMA-PET/CT.

12.4 Volume delineation

The extension of the region of interest (ROI) is delineated in the treatment-planning CT, with a co-registered MRI if applicable and with cognitive guidance alternatively co-registered with PSMA-PET/CT. A CT acquired at a different timepoint than the planning CT (e.g. the diagnostic PSMA-PET/CT) should also be evaluated for changes in ROI(s), see section 12.3.

12.4.1 Target volumes

The correctness of the delineated ROIs should be checked in axial, sagittal and coronal views. Tumour visible in radiologic imaging (MR/CT or PET) is considered the GTV.

For lymph nodes the entire node is delineated as GTV. If there are no signs of extranodal extension, the GTV=CTV.

For non-spine bony lesions, a clinical target volume (CTV) margin of 0 to 5 mm is considered appropriate according to international consensus (25, 26). The selected margin should reflect the level of image quality and anatomical certainty. When the gross tumour volume (GTV) is defined solely from PET uptake without a corresponding finding on CT or MRI, as in PSMA-RADS 4, or when the image resolution is limited, a margin of 5 mm is recommended to compensate for the higher degree of uncertainty. For spine lesions larger than 2 cm^3, two CTVs should be constructed. The first CTV is created by applying an isotropic margin of 0 to 5 mm to the GTV, using the larger margin if the delineation is based only on PET information. The second CTV should cover the relevant vertebral compartments in accordance with the system defined by the International Spine Radiosurgery Consortium (28, 29). Detailed guidance is provided in Section 12.10.2.1. For smaller spine lesions of 2 cm^3 or less, inclusion of vertebral compartments is not mandatory, and an isotropic margin of 0 to 5 mm to the GTV is considered sufficient. The larger margin is recommended in cases with a GTV based solely on PET- imaging, as described in Section 12.10.2.2.

In all cases where there is no soft-tissue extension, the CTV should be cropped to form an intraosseous CTV.

An ITV should be considered to adjust for target motion based on individual assessment of multiple image samples (see section 12.3).

The PTV margins specified in section 12.10.1-3 should provide a robust analysis for the minimum dose to the CTV and maximum dose to OAR. This robust analysis can be performed in the TPS by calculating the DVH uncertainties associated to target and/or OAR under displacements in x, y and z directions.

12.4.2 Organ(s) at risk

All relevant OAR(s) are to be delineated as they appear in the planning CT +/- co-registered MRI, in consistence with international guidelines (24). Serial OARs are to be delineated 1 cm cranial and caudal to the PTV. For critical serial OAR(s), e.g., nerve plexus or bowel, a Planning Organ at Risk Volume (PRV) expansion should be used. Highlighted details on delineation:

Spinal cord

For spinal lesions, a co-registered MRI is required to assess the extent of disease and position of the spinal cord. The spinal cord is to be defined for all target lesions that abuts the spinal canal from the base of scull to the caudal limit of L2. A PRV margin of minimum 2 mm for the spinal cord is mandatory for application of dose constraints. Additional margin could be appropriate according to patient related factors or uncertainties in the image registration. For target volumes outside of the columna, the spinal canal limited by bony anatomy is considered as PRV for the spinal cord.

Cauda Equina

The thecal sac is considered the PRV for Cauda Equina for which the dose constraints apply. The delineation of the thecal sac starts at the L2/L3 intersection and its caudal termination is at the level of the S1/S2 intersection. The entire bony canal is included to minimize variation in delineation and to include the filum terminale in accordance with the international consensus guidelines recommendations for sacral metastasis and the consensus for delineation of cauda equina published by Dunne et al (24-25). If the target prescription is compromised due to compromised PRV_CaudaEquina, the exact neural elements within the spinal canal as they appear on the planning CT and/or co-registered MRI can be contoured. A 2-3 mm isotrop margin is then used to construct the PRV_CaudaEquina.

Lumbar/Sacral plexus

The sacral plexus is delineated according to the recommendations by the UK-SABR consortium (26). One modification of the guidelines in the present protocol is the separation of the lumbar and sacral plexus if the target/s are positioned so that only one of the structures are needed. The plexus should be contoured to meet the contour of the cauda equina. The cranial aspect of the sacral plexus in the present protocol is the L5/S1 intersection starting at the first CT slice caudal to the cauda equina. A PRV margin of minimum 2 mm is mandatory for application of dose constraints.

Parallel OAR

For parallel OARs e.g., kidneys and liver, the entire organ must be delineated if it is located within 3 cm of the target.

12.5 Structure names in the treatment planning system

To upload treatment data, it is necessary to follow the naming structure in the present protocol for target volumes and OAR in accordance with the Swedish standard nomenclature in radiotherapy. All target volumes must be numbered. The number is followed by underscore and the prescribed dose (e.g., PTV1_30). All GTV/CTV incorporated in a PTV must be numbered accordingly and marked with the same dose (e.g., GTV1_30). The suggested approach is that each centre creates their own structure template, which should be applied to all enrolled patients. The responsibility to ensure the correctness of the data, including structure naming rests at each enrolling site.

12.6 Treatment planning

12.6.1 Beam characteristics

- Isocenter: one isocenter per plan (isocenter outside PTV is allowed)
- Angles: no restriction
- Couch rotation: allowed, under the provision of the local SBRT quality assurance (QA) protocol[5]
- Energies: no less than 6 MV, see 12.6.3
- Repetition rate: according to local SBRT QA protocol[5], be observant of high repetition rates in combination with large target motions, namely the interplay effect
- Jaw tracking: allowed, under the provision of the local SRT QA protocol[5]
- Minimum Field Size (jaws): a square equivalent 2 cm x 2 cm, under the provision of the local SBRT QA protocol[5]
- Technique: There is no restriction in treatment techniques. Static fields (3D-RT or IMRT), non-modulated arcs (ArcDynamic), and modulated arcs (VMAT) are allowed. Clinically acceptable plans with minimal complexity and treatment time are prioritised.
- The dosimetric effect of the immobilisation equipment and patients' limbs outside the FOV must be considered.

12.6.2 Dose-volume objectives

The prescribed total dose (D_P) in the present protocol is either 40 Gy in 5 fractions or 30 Gy in 3 fractions, depending on local practice and target location. In case of an inhomogeneous* dose prescription, the dose is prescribed to the periphery of the PTV, so that the prescribed isodose line of the maximum dose D_{max} encompasses the PTV. D98>95% of D_P, if the dose

[5] SRT QA protocol should cover the following aspects: the specific issues of the dosimetry of small fields, large dose per pulse and the mechanical QA of the linac internal (jaws, MLCs) and external (couch) accessories

constraints to critical OARs can be met. D_{max} should fall within the GTV and be approximately 125% or 150% of D_p for an 80% and 67% isodose prescription respectively.

The mean CTV dose should be approximately:

- 100-106% of prescribed dose for a 95% of D_{max} isodose prescription
- 110-120% of prescribed dose for an 80% of D_{max} isodose prescription*
- 130-140% of prescribed dose for a 67% of D_{max} isodose prescription*

12.6.3 Dose-volume constraints

The Normal Tissue Dose Constrains used in the present protocol follow the UK Consensus (27), which are un update and expansion from the previous American Association of Physicists in medicine (AAPM) task group 101 (28). Additional considerations/constraints in the present protocol:

- Mandatory D(0.1cc) to spinal cord < 26.5 Gy in 5 fractions and <21,5 Gy in 3 fractions apply.
- The spinal canal is replaced as PRV_SpinalCord by a 2 mm isotropic margin around the spinal cord for targets abutting the spinal canal (12.4.2).
- The D(0.1cc) to the spine should be restricted to ≤36 Gy to avoid fractures/vertebral compression using the 3 fraction-schedule (spine alt 2, section 12.10.2.2).

The most relevant constraints for the present protocol are summarised in Addendum I. If a study centre wishes to use another dose constraint than specified above or in Add. I, this must be documented and approved by QA-group before study entry.

It is strongly recommended not to exceed dose constraints. If a dose constraint cannot be met due to overlap of the target with an organ at risk, a lower isodose-prescription, or a switch from the 3-fraction schedule to the 5-fraction schedule can be used. Another option is to compromise the PTV coverage, so long as the analysed dose to the GTV is robust. In cases where the prescribed isodose or coverage of the PTV is compromised to meet the constraint, the $D_{98\%}$ delivered to the GTV should be at least the nominal dose in Table 1. **If these suggested alternatives are not sufficient to avoid exceeded dose constraints, the patient is to be taken out of the study as screen failure (exclusion criterion 8).** *All cases of dose reduction or target coverage compromise must be reported in the eCRF, as well as the resulting dose to the OAR*.
Note! Attention must be paid to all underdosed regions at the online CBCT matching.

12.6.4 Dose calculation

The planning will generally be done with 6 MV flattening filter free (FFF) photon beams. Type A algorithms[6] <u>*are disallowed*</u> in this study, as they base their inhomogeneity corrections on a mere path length scaling, ignoring lateral transport of electrons[3]. Hybrid algorithms (type A-B), like anisotropic analytical algorithm (AAA), are allowed. Type B algorithms[7], Monte Carlo and deterministic solvers (AcurosXB, Varian), are allowed.

---

[6] Modified Batho (Varian), Equivalent TAR (Varian), Pencil Beam Convolution (Varian, Oncentra/MasterPlan. XiO and PPLAN)
[7] Collapsed Cone (Pinnacle-Philips, Oncentra/MasterPlan), Collapsed Cone Superposition (XiO) and Collapsed Cone (Tomotherapy)

Beam energies higher than 6 MV are allowed, except for usage of AAA in targets surrounded by a low-density medium (e.g., lung). Further reading on dose calculation algorithms can be found at ICRU 91 (29) and Knöös *et al* (30) for example.

The dose distributions should be calculated with a grid size ≤ 2mm[5, 6].

12.7 Fractionation (scheduling)

Planning CT/MRI should be performed as soon as possible after randomisation to minimise the effect of TAB for target definition and IGRT. The first treatment should be given within 21 days after randomisation. For 30 Gy in 3 fractions MD-SBRT is given every other to every fourth day (>48 and <96 hours). For the 5-fraction schedule treatment should be given 1-3 days apart (>24 and <72 hours), but *never on more than two consecutive days*. A treatment session takes approximately 30–40 minutes, depending on the complexity of the treatment plan. If several targets are to be treated, they may generally be treated on the same day. However, there are two exceptions: firstly, when the prolonged treatment time makes it difficult for the patient to stay still in the treatment position, and secondly, when the treatment plans contribute with high summation doses to an organ at risk.

Dose recording and reporting shall be performed as described below in the QA section.

12.8 Online isocenter localisation

A volumetric technique for online isocenter localisation, like CBCT, is mandatory if treatment is delivered on a linear accelerator. An online pre-treatment CBCT is used to localise the planned isocenter by focusing the online match on the tumour position, and to check that the organs at risk have a configuration compatible with the scenario predicted by the CT used for treatment planning.

Post-treatment CBCTs are useful in cases where intrafraction base-line shifts are suspected. Three-dimensional correction by means of a kV or MV 2D/2D planar on-line match *are not allowed* in this protocol.

12.9 Adaptive planning

If a severe intrafraction base line shift is observed (e.g., by means of a post-treatment CBCT) a change in fractionation and a new plan may be required. The sum of the delivered fraction/s (initial plan) and the remaining fractions (new plan) must comply with the initial dose constraints and the effect in the target, for example with recalculation in equivalent dose in 2 Gy fractions (EQD2) or biologically iso-effective dose (BED).

12.10 SBRT treatment details

Table 1. Overview of fractionation schedules and target volumes by location

	Lymph nodes	Spine lesions alt 1	Spine lesions alt 2[8]	Non-spine bony lesions
8 Gy x 5	x	x	-	x
10 Gy x 3	x	-	x	x
GTV	Entire lymph node	Tumour	Tumour	Tumour
CTV	GTV+0-5mm	See section 12.10.2.1	GTV+0-5mm	GTV+0-5mm
ITV[11] *(optional)*	As needed to address motion, see section 12.3	As needed to address motion, see section 12.3	As needed to address motion, see section 12.3	As needed to address motion, see section 12.3
PTV[9]	CTV+5 mm	See section 12.10.2.1	CTV+3 mm	CTV+3-5 mm
Isodose prescription (of D_{max})	80%	See section 12.10.2.1	80%	80%
Max dose	125%	See section 12.10.2.1	125%	125%

12.10.1 Treatment details lymph nodes

In the case if multiple adjacent lymph nodes, they render separate target volumes only if their resulting PTVs do not overlap. Hence, multiple lymph nodes (separation <1 cm) can be treated (and counted) as one target for MD-SBRT. Due to the risk of adjacent OARs at risk of intra/inter-fraction deformation (rectum, bowel, bladder) special attention to individual image data sampled at different timepoints (section 12.4) is highly relevant when treating the pelvic lymph nodes.

Target volumes:

I. **GTV1-3_30*/40**** is defined as the whole lymph node in the planning CT with PET support and +/-co-registered MRI.
II. **CTV1-3_30*/40**** is constructed by adding a 0-5 mm isotropic margin around the GTV1-3_30*/40**, if no extranodal extension is suspected CTV=GTV.
III. **ITV1-3_30*/40**** is constructed as a separate ROI or incorporated in the CTV-PTV-margin as necessitated by inter- or intra-fractional movements in the individual image data (e.g., 4DCT or patient imaging data sampled at different time points).
IV. **PTV1-3_30*/40**** is constructed by adding a 5 mm isotropic margin to the CTV as a rule of thumb. Individual assessment of the setup errors is mandatory.

Dose prescription:

PTV_30*-40**: The dose is prescribed to the 80% isodose line encompassing the PTV, with target coverage such that PTV $D_{98\%} \geq 95\%$ of the prescription dose. This corresponds to a

[8] *GTV < 2cm^3*

[9] *Periodic movements are dealt with according to section 12.3.1 as either additional CTV-PTV-margin or a constructed ITV from the 4DCT.*

* 30 Gy in 3 fractions

** 40 Gy in 5 fractions

maximum dose of approximately 125%. If there is substantial distance to adjacent OARs, a 67% isodose prescription may be used, resulting in a maximum dose of about 150% of the prescription in the CTV centre. The mean CTV dose should be approximately 115% of the prescription dose (or 135% for a 67% isodose prescription). For small target volumes or cases with large expected intra-fraction motion, a 95% isodose prescription may be considered..

12.10.2 Treatment details spinal lesions

Target volumes are delineated in the treatment planning CT/co-registered T1 weighted MRI in planning position and doses are prescribed according to institutional practice as either alternative 1. or 2.

12.10.2.1 Alternative 1, spinal lesion:

Target volumes

I. **GTV1-3_40** comprises visual tumour lesion on a T1W MRI and/or a co-registered PET-CT/CT-scan. The correctness of the GTV delineation should be checked in axial, sagittal and coronal views.
II. **CTV1-3_40** is constructed by adding a 0-5 mm isotropic margin around the GTV. If no visible tumour in anatomical images (PSMA RADS 4) the larger margin is recommended. The CTV is cropped for anatomical borders and will not extend the limitation of the bone if no soft tissue component is present.
III. **CTV1-3_35** comprises the entire vertebra of the affected metastases excluding PTV1-3_40. If the tumor invades (or is close to) areas of the vertebra outside the vertebral body (pedicles, lamina, etc.), it is necessary to include an extra bony CTV margin for each case. These extra margins may encompass the pedicle, lamina, transverse process or spinous process, according to international guidelines (31, 32).
IV. **PTV:** Two PTVs are used for plan evaluation, recommended nomenclature is PTV1-3_ 40 and PTV1-3_35. Definition of these structures are:
    1. **PTV1-3_40**: 1 mm transversal (2 mm for very small GTVs) and 2mm cranio-caudal expansion from CTV1-3_40
    2. **PTV1-3_35**[10]: 2 mm transversal and 3 mm craniocaudal expansion from CTV1-3_35, excluding PTV1-4_40+1mm

Dose prescription

I. **PTV1-3_35**: A total dose of 35 Gy is prescribed to PTV_{35}, with target coverage such that PTV $D_{98}\%$ ≥ 98% of the prescription dose. The dose within the PTV should not exceed 106% of the prescribed dose, and the mean dose to CTV_{35} should be approximately 35.5 Gy, kept as homogeneous as possible.
II. **PTV1-3_40:** The prescription dose is 40 Gy to PTV_{40}, with target coverage such that PTV $D_{98}\%$ ≥ 95% of the prescription dose. Dose within the PTV should range between 95% and 110% of the prescribed dose (38–44 Gy). The mean dose to CTV_{40} should be between 40 Gy and 41 Gy.

[10] PTV_GTV_40 must be cropped from PTV_CTV_35

12.10.2.2 *Alternative 2, spinal lesion GTV<2cm^3:*

Target volumes

I. **GTV1-3_30** comprises visual tumour lesion on a T1W MRI and/or a co-registered PET-CT/CT . The correctness of the structure delineation should be checked in axial, sagittal and coronal views.

II. **CTV1-3_30** is constructed by adding an isotropic margin of 0-5 mm. If the GTV is delineated based on PSMA-PET/CT without visible disease in MRI/CT (PSMA RADS 4) the larger margin is applied. The CTV is cropped for anatomical borders and will not extend the limitation of the bone if no soft tissue component is present.

III. **PTV1-3_30** is constructed by adding a 2 mm transversal and 3 mm craniocaudal expansion from CTV1-3_30. Individual assessment of the setup errors is mandatory.

Dose prescription

A total dose of 30 Gy is prescribed to the PTV, corresponding to the 80% isodose line. Target coverage shall be achieved such that PTV D_{98}% ≥ 95% of the prescription dose. This results in a maximum dose of approximately 125% of the prescription (D_{120}% ≤ 1 cc) and a mean CTV dose of about 115%. If a 95% isodose prescription is used instead, the corresponding Dmax is approximately 105% and the mean CTV dose about 100%..

12.10.3 Treatment details non-spinal bony lesions

Target volumes

I. **GTV1-3_30[*]-40[**]** is defined as all visible tumour including suspected diffuse growth at border. The correctness of the structure delineation should be checked in axial, sagittal and coronal views. The 3-fraction schedule is not recommended outside the spine and pelvis.

II. **CTV1-3_30[*]-40[**]** is constructed by adding an isotropic margin of 0-5 mm. If no visible tumour in anatomical images (PSMA RADS 4) the larger margin is recommended. The CTV is cropped for anatomical borders and will not extend the limitation of the bone if no soft tissue component is present.

III. **PTV1-3_30[*]-40[**]** is constructed by adding a 3-5 mm isotropic margin around the CVT1-3_40. Individual assessment of the setup errors is mandatory.

Dose prescription

PTV1-3_30[*]40[**]: The dose is prescribed to the 80% isodose line, with target coverage such that PTV D_{98}% ≥ 95% of the prescription dose. This corresponds to a maximum dose of approximately 125% and a mean CTV dose of about 115%. In cases with a soft-tissue tumoral component protruding beyond bone, a 67%-isodose prescription may be used, provided no OARs are in close proximity and the Dmax is located within the soft-tissue component. For small target volumes or cases with large expected intra-fraction motion, a 95%-isodose prescription may be considered. For PTVs adjacent to lung or other low-density regions, coverage objectives should be robustly evaluated to ensure adequate CTV coverage rather than relying solely on nominal PTV coverage.

---

[*] 30 Gy in 3 fractions
[**] 40 Gy in 5 fractions

12.11 Radiotherapy, quality assurance (RT-QA)

The purpose of the QA protocol is to ensure uniformity of all radiotherapy data for all patients.
A QA-group will be assigned to collect treatment and verification data, including documentation on set up and immobilisation prior to each centres study entry to ensure compliance to the study protocol.
MD-SBRT related treatment information and other relevant documentation for each patient shall be uploaded according to AD II at completion of MD-SBRT. Dose reporting will be done in accordance with the ICRU report 91 on prescribing, recording, and reporting of stereotactic treatments with small photon beams (28). Patient data stored digitally in the DICOM format, such as CT-images, dose plans, and dose distributions, should be reported according to AD. II.
Dummy runs, including both delineation of structures and treatment planning will be performed. When ten patients are included in the intervention arm, structure delineation and dose-volume constraints/objectives will be evaluated and discussed in the study group. Quality audits may be performed.
To maintain the quality of the treatment, each participating centre should strive to include a minimum of 5 patients per year. If a centre includes less than 2 patients per year, this centre should be considered for closing.

12.12 Dummy run procedure

A dummy run is scheduled before the start of the patient trial. PSMA-PET/CT and MR images of two oligometastatic cancer patient will be sent to participating centres from the QA group. Delineation of target volumes and OARs according to the study protocol will be performed at each centre, as well as a treatment plan according to the study protocol. Evaluation of target delineation and treatment plans will be performed by the steering committee and QA group before the centre starts to include patients.

12.13 Individual patient checks

A detailed description how to prepare and process the data needed for the QA process is presented in AD. II.

13 Patient reported outcome (PRO) and health related QoL

The QLQ will be a main source for the assessment of treatment related side effects and health related QoL. EORTC QLQ-C30 (version 3.0) is patient-based and designed for self-administration. The cross-cultural validity and the psychometric properties are considered satisfactory. It has previously been used in QoL studies of prostate cancer. It measures functions, symptoms, and global health.
Assessments will be made at randomisation, and on four more occasions (3, 12, 36 and 60 months after randomisation) during the study.

14 Statistical plan

14.1 Sample size determination

The primary efficacy endpoint used for the power calculation is FFS defined as the time from randomization to biochemical failure. A median FFS of 36 months for low volume de novo prostate cancer patients diagnosed with conventional radiology is suggested in the literature

without the addition of abiraterone (14). In the present scenario with 2 years of abiraterone, PSMA-PET/CT-staging and a proportion of patients with recurrent HSPC, we anticipate a median FFS of 72 months (16). Based on previous trials of MD-SBRT a 50% reduction of treatment failures for the intervention group compared to the control group representing a hazard ratio of 0.5 is expected (6, 7). A sample size assuming failure times follows an exponential distribution, using a 1:1 randomization scheme (MD-SBRT+ standard treatment:standard treatment), 80% power, type I error of 5% (one-sided), withdrawal probability of 5% and based on a log-rank-test gives 52 events from 118 patients. Accrual time for recruitment of the patients is estimated to be 3 years, and the calculation is based on that patients will be followed up for a minimum of 6 years.

14.2 Statistical methods and analysis plan

The primary analysis on bPFS will be performed on the intention-to-treat analysis set (see below), but analysis will also be performed based on actual received treatment. bPFS is defined for each patient as the time between randomisation and the time of biochemical failure. All eligible follow-up time for each patient will be used in the analysis. If a patient expires without recurrence, the patient will be censored at that date. Every effort will be made to collect endpoint data on subjects, including subjects withdrawn from treatment for any reason, who are eligible to participate in the study and who have not withdrawn consent for survival data collection. If a patient has a registered treatment failure at first follow up after treatment, the time of failure will be defined as the randomisation date.
The primary endpoint (bPFS) will be analysed after pre-specified number of events have occurred. bPFS will be analysed using a Cox proportional hazards regression model. The proportional hazards assumption on the stratification variables will be evaluated by testing the association between Schoenfeld residuals and follow-up time. In the event of non-proportional hazards, stratification variables will be incorporated into the model as strata.
For the primary endpoint (bPFS), a treatment-policy strategy will be applied: participants will remain in the risk set regardless of intercurrent events such as treatment discontinuation, or changes in background standard of care, and progression events will be defined according to the defined criteria.

If baseline characteristics differ between treatment groups, sensitivity analyses will be conducted by adjusting for these variables in multivariable Cox regression models. Kaplan–Meier survival curves will be generated to visualize the distribution of bPFS and other survival endpoints by treatment group.

The Mann-Whitney U-test or Fishers exact test will be used for safety analysis. An interim analysis of acute toxicity will be made after median follow up of 6 months. Safety analysis of late toxicity will be analysed after study closure.

14.3 Subject populations(s) for analysis

Intention to treat population: Any subject randomised in the study, regardless of whether they received study treatment.
Per protocol population: Any subject who has undergone required protocol processing.

15 Safety reporting

15.1 Definitions of Serious Adverse Event (SAE)

Serious Adverse Event (SAE) is an adverse event that:

- results in death
- is life-threatening
- requires inpatient hospitalisation or prolongation of existing hospitalisation
- results in persistent or significant disability/incapacity
- is a congenital anomaly/birth defect

or

- toxicity according to CTCAE v5 grade 4-5

15.2 Reporting procedure

All AEs grade 3 and above according to CTCAE v5.0 are to be reported in the eCRF, including causality assessment.
AEs of all grades must be reported for all organs within 3 cm of the irradiated volume(s) for 60 months to detect late side effects after MD-SBRT. Side effects that are suspected to be related to the study intervention, MD-SBRT, should be reported and graded according to CTCAE v5.0 using CTC terminology and noted in the eCRF in the Toxicity module. This is suggested to be carried out through collection of toxicity from responsible care unit every 6th month by delegated study personnel.

- All SAEs with possible causality with the delivered MD-SBRT must be sent to the Data Centre via the eCRF within 24 hours after notification by the study personnel. The Data Centre will notify the steering committee. All SAEs and any actions taken will be noted in the protocol of the scheduled study meetings.
  Note! SAEs with possible causality with the delivered MD-SBRT must be reported to Data Centre during 60 months of follow up, even if CRPC progression is registered.

16 Quality assurance

For quality assurance of radiotherapy, see above.

16.1 Control of data consistency

All patients included in the study are identified by the patient identification number. Subject identification code lists, that links patients' names to the patients' identification number, must be stored in the Investigator File, securely and inaccessible to unauthorised persons. Study data will be recorded via electronic Case Report Forms (eCRF). Study data may be recorded directly into the eCRF, i.e., the eCRF may be the source data, or be transcribed by the site from the paper source documents onto the eCRF according to the local source-data list. Prior to study start, the Investigator and the Monitor must identify and document the expected source locations for the data collected in the study. Expected source locations are for example the subject's medical record, laboratory reports and the eCRF itself.
Accurate and reliable data collection will be assured by verification of the eCRF against the investigator's records and medical records by a study monitor, as well as study integrity, compliance with the protocol and applicable regulations.

16.2 Site monitoring

Monitoring is performed as per the study's monitoring plan and there will be regular contacts with the study sites to verify that the:

- Data are authentic, accurate, and complete.
- Safety and rights of subjects are being protected.
- Trial is conducted in accordance with the currently approved protocol and any other trial agreements and all applicable regulatory requirements.

16.3 Legal aspects

Investigators of participating centres agree to co-operate with any quality assurance visit undertaken by third parties.
The study will be performed after approval of the Swedish Ethical Review Authority and, where applicable, other national ethical review authorities. Essential changes in the protocol must be communicated with the Swedish Ethical Review Authority and to other relevant authorities as applicable.

16.4 Direct access to data/documents

The sponsor has the responsibility to maintain adequate and accurate records to enable the conduct of the study to be fully documented and the study data to be subsequently verified. Investigators files and subjects clinical source documents must be kept after completion or discontinuation of the study according to Swedish laws and regulations.

17 Data management

17.1 Patient registration/randomisation procedure

Subject eligibility will be established before treatment randomisation. Subjects will be randomised strictly sequentially, as subjects are eligible for enrolment/randomisation.
If a subject discontinues from the study, the subject number will not be reused, and the subject will not be allowed to re-enter the study. When a patient is found to be eligible and has signed a written informed consent, randomisation will be performed via the eCRF.

17.2 Case report forms

Study data will be recorded via the eCRF. The eCRF should be filled in at each time point for follow-up.
The investigator at the enrolling site is responsible for collection of source data if patient visits are decentralised to other clinics.

17.3 Health related quality of life forms - PRO

QLQ (EORTC QLQ-C30 (version 3.0)) will be administered to the patient on five occasions during the study period:

- before start of treatment.
- at follow-up at three months, one year, three and five years after randomisation.

The QLQ can be completed digitally and automatically uploaded to the eCRF or collected manually by mail or at study visit by the study coordinator. The first QLQ at baseline must be

filled out before randomisation, preferably at site. If needed, the following questionnaires can be filled out at the hospital in connection to the scheduled follow up visits.

17.4 Imaging and RT data

The diagnostic PSMA-PET/CT can be uploaded according to Addendum II after IC as part of the screening procedure (see section 10.1). For simplicity, it is suggested that the diagnostic PSMA-PET/CT is uploaded by the same identified study personnel that upload the MD-SBRT-data, if possible. The MD-SBRT-data is uploaded according to AD. II at the end of MD-SBRT. The PSMA-PET/CT performed at progression should be uploaded according to AD. II at end of study visit (see section 10.10).
It is recognized that other workflows may be necessary to adhere to national and local data-regulations, but in such cases, this should be stipulated at study start for each study centre.

18 Exploratory biomarkers

18.1 Blood biomarkers

For patients recruited to the blood biomarker part of the present project at Umeå University hospital, additional plasma will be sampled and used for isolation of both CTCs and platelets. Both have both been proven to contain tumor-derived biomarkers of therapy-predictive value in patients with metastatic prostate cancer (33, 34) and will be evaluated as liquid biopsies to monitor tumor characteristics before and during therapy as well as to assess their potential as biomarkers for patient selection for MD-SBRT in oligometastatic HSPC. All analyses will be performed both for the ITT population and separated according to strata in the second blood biomarker cohort as well. Given the results of a pre-planned futility analysis in this cohort (first 10 patients), additional centers will be invited to include also in the second phase blood biomarker sub-study.
All patients must sign additional informed consent before collection of samples for blood biomarker analysis.

18.2 PSMA-PET biomarkers

Imaging parameters from the diagnostic ^{68}Ga/^{18}F-PSMA-PET/CT will be assessed for association with the analysed survival outcomes. For advanced castration-resistant prostate cancer, limited number of studies have shown PSMA parameters mean standardised uptake value (SUV_{mean}) (35) and maximum standardised uptake value (SUV_{max}) ratios (lesion/spleen and lesion/liver) (36), were associated with lower survival rates. A small study of patients undergoing ^{223}Ra therapy showed that PSMA+ functional tumour volume (FTV) may have the potential to predict treatment response (37). For hormone-sensitive prostate cancer or other treatment stages of prostate cancer, the prognostic value of PSMA-PET/CT has not been studied, to our knowledge.
In the identification of PET biomarkers for other prostate cancer tracers, such as ^{11}C-choline, FTV and uptake volume product or the equivalent “total lesion glycolysis” (TLG) have been found to predict shorter progression-free survival in primary high-risk prostate cancer patients (38). For ^{11}C-acetate, PET positivity has been shown to predict treatment failure in intermediate- and high-risk prostate cancer, and lower survival in biochemical relapse, where SUV_{max} and uptake volume product of total ^{11}C-acetate positive tumour burden correlated with time-to-death (39, 40).Based on this knowledge and on the conditions of the

study design, we will quantify baseline PSMA-PET parameters SUV_{max}, SUV_{mean}, total FTV and total TLG, to investigate their prognostic value and possible cut-offs. Additionally, we will score lesion uptakes relative to physiological background activity in blood pool, liver and parotid glands, according to the standardised PROMISE classification (41), as well as the PSMA-RADS classification (10) and evaluate the prognostic implication of the highest score for this patient group.
At progression the same PSMA-PET parameters will be evaluated, with addition of delta (change in) SUV_{max}, SUV_{mean}, total FTV, total TLG and highest PROMISE and PSMA-RADS score. Furthermore, PSMA-PET evaluated patterns of failure will be evaluated to assess the effect of MD-SBRT, and to explore possibilities for further treatment.

19 Ethical considerations

19.1 Patient protection

The study is to be performed in accordance with the ethical recommendations of the Helsinki declaration, or the laws and regulations of the country, whichever provides the greatest protection of the patient.

19.2 Patient integrity

The investigator must assure that the patient’s anonymity will be maintained and that their identities are protected from unauthorised parties. On CRFs or other documents submitted to the Data Centre patients should only be identified by their identification code, year and date of birth.

19.3 Informed consent

All subjects will receive written and oral information about the aims of the study, possible hazards, and the mechanism of treatment allocation. They will be informed about the strict confidentiality of their patient data, but that their medical records may be reviewed for trial purposes by authorized individuals other than their treating physician.
It will be emphasized that the participation is voluntary and that the patient is allowed to decline further participation whenever he/she wants. If the patient wishes to withdraw from the study, he or she will be offered the standard treatment at the clinic.
A signed, informed consent must be obtained from the patient before study entry. In the cases were the patient declines to participate in the study the conventional treatment according to local practice should be offered.
If new safety information results in significant changes in the risk/benefit assessment, the consent form should be reviewed and updated if necessary. All patients (including those already being treated) should be informed of the new information, given a copy of the revised form and give their consent to continue in the study.

19.4 Independent ethical review

Applicable details from this protocol and all accompanying material provided to the patient, such as written patient information used to obtain informed consent, has been submitted by the investigator to the Swedish Ethical Review Authority. Approval from the authority has been granted 2021-06-22 (dnr 2021-02766), amended 2023-03-07 (dnr 2023-01224-02),

2023-07-19 (dnr 2023-04372-02), 2024-06-05 (dnr 2024-02744-02) and 2025-03-18 (dnr 2025-01461-02).

19.5 Risk benefit analysis

Attempts at disease control with MD-SBRT is already in use for highly selected cases, and not available to all patients. The optimal patient selection for the intervention, potential gain and toxicity of this treatment is still uncertain. The present project is an important addition in personalising the treatment for prostate cancer. Results from MD-SBRT in other cancer diagnoses have provided stabile remission for a number of patients and thus been an important advance for survival endpoints. The use of more sensitive diagnostic imaging, PSMA-PET/CT has introduced a stage migration that possibly excludes patients from treatment with a curative intent. The patient cohort in the present trial are certainly represented in the cohorts of high-risk prostate cancer treated with curative intent in today's literature. We therefore investigate the potential of treating all known disease to ablative doses in a multi-centre randomised phase III-study in relation to clinically relevant endpoints (38). Treatment outside of the present trial is lifelong ADT +/- lifelong 2nd generation hormonal agents and chemotherapy associated with increasing pharmaceutical and health care costs as well as systemic side effects for the patient.
Today, the only selection of patients for the study intervention is counting metastatic lesions. The present trial will attempt to link possible biomarkers to outcome and treatment response in blood and image biomarkers to improve patient selection, a potential benefit for both non-responders and responders to the study intervention.
Besides the research questions, the METRO-collaboration also provides a platform for national collaboration in advanced radiotherapy that can be utilised for future research in the field as well as implementation of novel treatment techniques.

20 Participating centres

All hospitals fulfilling the QA-requirements for the SBRT and the minimal patient inclusion will be invited to take part in the study. Each centre must expect to include a minimum of 5 patients per year. If a centre includes less than 2 patients per year, that centre should be considered for closing. Previously included patients will then be followed according to the protocol but no further inclusion is accepted from that centre.
Collection of blood samples for analysis of biomarkers will be performed after additional informed consent.

21 Ownership of data

The data is owned by the Study Group consisting of the investigators at the participating centres and collaborators in blood and imaging biomarkers. The data of an individual centre/principal for biomarkers cannot be extracted for publications to answer the questions of this trial. Future research projects utilising data from the present trial must be approved by the study group.

21.1 Meetings

Regular meetings with the Study Group (and other representatives from participating centres) will be held twice per year or when considered necessary. The Study Steering

Committee including principals for imaging and blood biomarkers will also have separate meetings when necessary. In the time between Study Group meetings, the principal investigator shall act as contact person and will have an executive role, with the aid of the Study Steering Committee. Decisions taken shall be discussed at the meetings of the Study Group. The principal investigator is responsible to arrange the meetings of the Study Group/ Study Steering Committee.

21.2 Reporting

All presentations of data from the study should only be made after agreement within the steering committee. The results of the study will be submitted to an internationally recognised scientific medical journal. Apart from the Steering committee, each participating centre with at least 10 patients included will be guaranteed co-authorship for one person. In addition to this, persons with special responsibilities within the study may become co-authors. The Vancouver declaration will be followed in all publications based on this study. Collaborators in blood-biomarkers are given due time to protect arising intellectual property after notice of planned dissemination activity.

22 Flow chart of study procedures

	Screening	Baseline	1 month	End of MD-SBRT[11]	3 months after rand.	6 months after rand.	Every 3rd month[12]	Every 6th month[13]	Year 1, 3 and 5	Disease progression
Visit window (days)	0 (0/-1)	0 (+5)	30 (+/-14)	30 (+/-14)	90 (+/-14)	180 (+/-21)	(+/-21)	(+/-30)	(+/-30)	(+/-21)
Incl./excl criteria	X									
Informed consent	X									
QLQ-C30		X			X				X	
Medical history including prostate cancer		X								
Physical examination		X			X					X
Haematology and blood chemistry		X		X[14]		X[16]		X[16]	X[16]	X[16]
Randomisation	X									
Biochemical control					X	X	X	X	X	X
Side effects, CTCAE: Arm A		X		X	X	X		X	X	
Reporting of RT-data				X						
Reporting of SOC					X				X[15]	
Adverse Event >/=grade 3			X		X	X		X	X	X
CT/MRI/PET	X[16]									X
Blood biomarkers		X	X		X					X
Initiation of ADT		X								

[11] Arm A (can be performed at 1 month visit)
[12] Until progression CRPC
[13] Until 60 months in Arm A, until progression CRPC or 60 months in Arm B
[14] As needed to assess side effects (Arm A)
[15] At 12, 24 and 36 months
[16] PSMA-PET/CT approx. within 30 days before informed consent

23 References

1. Hellman S, Weichselbaum RR. Oligometastases. Journal of clinical oncology : official journal of the American Society of Clinical Oncology. 1995;13(1):8-10.
2. Ruers T, Van Coevorden F, Punt CJ, Pierie JE, Borel-Rinkes I, Ledermann JA, et al. Local Treatment of Unresectable Colorectal Liver Metastases: Results of a Randomized Phase II Trial. Journal of the National Cancer Institute. 2017;109(9).
3. Lodeweges JE, Klinkenberg TJ, Ubbels JF, Groen HJM, Langendijk JA, Widder J. Long-term Outcome of Surgery or Stereotactic Radiotherapy for Lung Oligometastases. J Thorac Oncol. 2017;12(9):1442-5.
4. Hadden WJ, de Reuver PR, Brown K, Mittal A, Samra JS, Hugh TJ. Resection of colorectal liver metastases and extra-hepatic disease: a systematic review and proportional meta-analysis of survival outcomes. HPB (Oxford). 2016;18(3):209-20.
5. Palma DA, Olson R, Harrow S, Gaede S, Louie AV, Haasbeek C, et al. Stereotactic ablative radiotherapy versus standard of care palliative treatment in patients with oligometastatic cancers (SABR-COMET): a randomised, phase 2, open-label trial. Lancet. 2019;393(10185):2051-8.
6. Phillips R, Shi WY, Deek M, Radwan N, Lim SJ, Antonarakis ES, et al. Outcomes of Observation vs Stereotactic Ablative Radiation for Oligometastatic Prostate Cancer: The ORIOLE Phase 2 Randomized Clinical Trial. JAMA Oncol. 2020;6(5):650-9.
7. Ost P, Reynders D, Decaestecker K, Fonteyne V, Lumen N, De Bruycker A, et al. Surveillance or Metastasis-Directed Therapy for Oligometastatic Prostate Cancer Recurrence: A Prospective, Randomized, Multicenter Phase II Trial. J Clin Oncol. 2018;36(5):446-53.
8. Lievens Y, Guckenberger M, Gomez D, Hoyer M, Iyengar P, Kindts I, et al. Defining oligometastatic disease from a radiation oncology perspective: An ESTRO-ASTRO consensus document. Radiotherapy and oncology : journal of the European Society for Therapeutic Radiology and Oncology. 2020;148:157-66.
9. Hofman MS, Lawrentschuk N, Francis RJ, Tang C, Vela I, Thomas P, et al. Prostate-specific membrane antigen PET-CT in patients with high-risk prostate cancer before curative-intent surgery or radiotherapy (proPSMA): a prospective, randomised, multicentre study. Lancet. 2020;395(10231):1208-16.
10. Rowe SP, Pienta KJ, Pomper MG, Gorin MA. Proposal for a Structured Reporting System for Prostate-Specific Membrane Antigen-Targeted PET Imaging: PSMA-RADS Version 1.0. J Nucl Med. 2018;59(3):479-85.
11. Rowe SP, Pienta KJ, Pomper MG, Gorin MA. PSMA-RADS Version 1.0: A Step Towards Standardizing the Interpretation and Reporting of PSMA-targeted PET Imaging Studies. Eur Urol. 2018;73(4):485-7.
12. Finianos A, Gupta K, Clark B, Simmens SJ, Aragon-Ching JB. Characterization of Differences Between Prostate Cancer Patients Presenting With De Novo Versus Primary Progressive Metastatic Disease. Clin Genitourin Cancer. 2017.

13. Nizialek E, Lim SJ, Wang H, Isaacsson Velho P, Yegnasubramanian S, Antonarakis ES. Genomic profiles and clinical outcomes in primary versus secondary metastatic hormone-sensitive prostate cancer. Prostate. 2021;81(9):572-9.
14. Parker CC, James ND, Brawley CD, Clarke NW, Hoyle AP, Ali A, et al. Radiotherapy to the primary tumour for newly diagnosed, metastatic prostate cancer (STAMPEDE): a randomised controlled phase 3 trial. Lancet. 2018;392(10162):2353-66.
15. Ferro M, Lucarelli G, Crocetto F, Dolce P, Verde A, La Civita E, et al. First-line systemic therapy for metastatic castration-sensitive prostate cancer: An updated systematic review with novel findings. Crit Rev Oncol Hematol. 2021;157:103198.
16. Attard G, Murphy L, Clarke NW, Cross W, Jones RJ, Parker CC, et al. Abiraterone acetate and prednisolone with or without enzalutamide for high-risk non-metastatic prostate cancer: a meta-analysis of primary results from two randomised controlled phase 3 trials of the STAMPEDE platform protocol. Lancet. 2022;399(10323):447-60.
17. Supiot S, Vaugier L, Pasquier D, Buthaud X, Magné N, Peiffert D, et al. OLIGOPELVIS GETUG P07, a Multicenter Phase II Trial of Combined High-dose Salvage Radiotherapy and Hormone Therapy in Oligorecurrent Pelvic Node Relapses in Prostate Cancer. Eur Urol. 2021;80(4):405-14.
18. Roach M, 3rd, Hanks G, Thames H, Jr., Schellhammer P, Shipley WU, Sokol GH, et al. Defining biochemical failure following radiotherapy with or without hormonal therapy in men with clinically localized prostate cancer: recommendations of the RTOG-ASTRO Phoenix Consensus Conference. Int J Radiat Oncol Biol Phys. 2006;65(4):965-74.
19. Morgan TM, Boorjian SA, Buyyounouski MK, Chapin BF, Chen DYT, Cheng HH, et al. Salvage Therapy for Prostate Cancer: AUA/ASTRO/SUO Guideline Part I: Introduction and Treatment Decision-Making at the Time of Suspected Biochemical Recurrence after Radical Prostatectomy. J Urol. 2024;211(4):509-17.
20. Widmark A, Gunnlaugsson A, Beckman L, Thellenberg-Karlsson C, Hoyer M, Lagerlund M, et al. Ultra-hypofractionated versus conventionally fractionated radiotherapy for prostate cancer: 5-year outcomes of the HYPO-RT-PC randomised, non-inferiority, phase 3 trial. Lancet. 2019;394(10196):385-95.
21. Dearnaley D, Syndikus I, Mossop H, Khoo V, Birtle A, Bloomfield D, et al. Conventional versus hypofractionated high-dose intensity-modulated radiotherapy for prostate cancer: 5-year outcomes of the randomised, non-inferiority, phase 3 CHHiP trial. The Lancet Oncology. 2016;17(8):1047-60.
22. Michalski JM, Lawton C, El Naqa I, Ritter M, O'Meara E, Seider MJ, et al. Development of RTOG consensus guidelines for the definition of the clinical target volume for postoperative conformal radiation therapy for prostate cancer. International journal of radiation oncology, biology, physics. 2010;76(2):361-8.
23. Sidhom MA, Kneebone AB, Lehman M, Wiltshire KL, Millar JL, Mukherjee RK, et al. Post-prostatectomy radiation therapy: consensus guidelines of the Australian and New Zealand Radiation Oncology Genito-Urinary Group.

Radiotherapy and oncology : journal of the European Society for Therapeutic Radiology and Oncology. 2008;88(1):10-9.
24. Poortmans P, Bossi A, Vandeputte K, Bosset M, Miralbell R, Maingon P, et al. Guidelines for target volume definition in post-operative radiotherapy for prostate cancer, on behalf of the EORTC Radiation Oncology Group. Radiotherapy and oncology : journal of the European Society for Therapeutic Radiology and Oncology. 2007;84(2):121-7.
25. Nguyen TK, Louie AV, Kotecha R, Saxena A, Zhang Y, Guckenberger M, et al. Stereotactic body radiotherapy for non-spine bone metastases: A meta-analysis and international stereotactic radiosurgery society (ISRS) clinical practice guidelines. Radiother Oncol. 2025;205:110717.
26. Nguyen TK, Chin L, Sahgal A, Dagan R, Eppinga W, Guckenberger M, et al. International Multi-institutional Patterns of Contouring Practice and Clinical Target Volume Recommendations for Stereotactic Body Radiation Therapy for Non-Spine Bone Metastases. Int J Radiat Oncol Biol Phys. 2022;112(2):351-60.
27. Hanna GG, Murray L, Patel R, Jain S, Aitken KL, Franks KN, et al. UK Consensus on Normal Tissue Dose Constraints for Stereotactic Radiotherapy. Clinical oncology (Royal College of Radiologists (Great Britain)). 2018;30(1):5-14.
28. Benedict SH, Yenice KM, Followill D, Galvin JM, Hinson W, Kavanagh B, et al. Stereotactic body radiation therapy: the report of AAPM Task Group 101. Med Phys. 2010;37(8):4078-101.
29. Measurements. ICoRUa. ICRU Report 91. 2014;Journal of the International Commission on Radiation Units and Measurements(14(2)):1–160.
30. Knöös T, Wieslander E, Cozzi L, Brink C, Fogliata A, Albers D, et al. Comparison of dose calculation algorithms for treatment planning in external photon beam therapy for clinical situations. Phys Med Biol. 2006;51(22):5785-807.
31. Cox BW, Spratt DE, Lovelock M, Bilsky MH, Lis E, Ryu S, et al. International Spine Radiosurgery Consortium consensus guidelines for target volume definition in spinal stereotactic radiosurgery. Int J Radiat Oncol Biol Phys. 2012;83(5):e597-605.
32. Dunne EM, Sahgal A, Lo SS, Bergman A, Kosztyla R, Dea N, et al. International consensus recommendations for target volume delineation specific to sacral metastases and spinal stereotactic body radiation therapy (SBRT). Radiother Oncol. 2020;145:21-9.
33. Josefsson A, Larsson K, Freyhult E, Damber JE, Welén K. Gene Expression Alterations during Development of Castration-Resistant Prostate Cancer Are Detected in Circulating Tumor Cells. Cancers (Basel). 2019;12(1).
34. Tjon-Kon-Fat LA, Lundholm M, Schröder M, Wurdinger T, Thellenberg-Karlsson C, Widmark A, et al. Platelets harbor prostate cancer biomarkers and the ability to predict therapeutic response to abiraterone in castration resistant patients. Prostate. 2018;78(1):48-53.
35. Ferdinandus J, Violet J, Sandhu S, Hicks RJ, Ravi Kumar AS, Iravani A, et al. Prognostic biomarkers in men with metastatic castration-resistant prostate cancer receiving [177Lu]-PSMA-617. Eur J Nucl Med Mol Imaging. 2020;47(10):2322-7.

36. Komek H, Can C, Yilmaz U, Altindag S. Prognostic value of 68 Ga PSMA I&T PET/CT SUV parameters on survival outcome in advanced prostat cancer. Ann Nucl Med. 2018;32(8):542-52.
37. Michalski K, Ruf J, Goetz C, Seitz AK, Buck AK, Lapa C, et al. Prognostic implications of dual tracer PET/CT: PSMA ligand and [(18)F]FDG PET/CT in patients undergoing [(177)Lu]PSMA radioligand therapy. Eur J Nucl Med Mol Imaging. 2021;48(6):2024-30.
38. Tseng JR, Yang LY, Lin YC, Liu CY, Pang ST, Hong JH, et al. Metabolic Volumetric Parameters in (11)C-Choline PET/MR Are Superior PET Imaging Biomarkers for Primary High-Risk Prostate Cancer. Contrast Media Mol Imaging. 2018;2018:8945130.
39. Haseebuddin M, Dehdashti F, Siegel BA, Liu J, Roth EB, Nepple KG, et al. 11C-acetate PET/CT before radical prostatectomy: nodal staging and treatment failure prediction. J Nucl Med. 2013;54(5):699-706.
40. Regula N, Häggman M, Johansson S, Sörensen J. Malignant lipogenesis defined by (11)C-acetate PET/CT predicts prostate cancer-specific survival in patients with biochemical relapse after prostatectomy. Eur J Nucl Med Mol Imaging. 2016;43(12):2131-8.
41. Eiber M, Herrmann K, Calais J, Hadaschik B, Giesel FL, Hartenbach M, et al. Prostate Cancer Molecular Imaging Standardized Evaluation (PROMISE): Proposed miTNM Classification for the Interpretation of PSMA-Ligand PET/CT. J Nucl Med. 2018;59(3):469-78.

Version	Date	Change in protocol	In protocol	Responsible
1	2021-06-22	Ethics aproval, dnr 2021-02766.		KS
2<br>AD III	2022-04-27	Clarification on PSMA-PET/CT	Addendum II	KS, SS (radiology)
3<br>Additional research party (iCellate AB, St Göran), data management CMRAD.<br>Ethics aproval, dnr 2023-01224-02.	2023-03-13		12.11, 16.4, 17.4,18.1, 19.4,22<br><br>Addendum II (revised), IV (new)	KS, iCellate, JJ
4<br>Change in Standard treatment (time limit on ADT and addition of abiraterone). New power calculation and randomization list. Ethics approval dnr 2023-04372-02.	2023-07-31	All chapters revised except: 9,13,16,18,20-21		KS, OB
4.1	2023-10-07	Clarification of Patient information: Expected minimum follow up time 60 months, Specified in protocol Ch. 7 Study period.	Clarification of Patient information and in protocol p.15	KS, IL
4.2	2023-10-23	22, footnote 17 and Reporting of Standard Treatment. Column to track changes in version history	Page 35	KS
4.3	2023-12.28	Minor adjustment 22 and adjusted 10 to reflect additional information in eCRF regarding new standard treatment.	Page 17, 35	KS

5<br><br>Change in exclusion criteria 6 and 7: Regional lymph node metastases are not counted as ground for exclusion based on number of metastases, ethics approval dnr 2024-02744-02.	2024-06-12	Study intervention and Standard treatment: Regional lymph nodes are primarily treated with standard pelvic lymph node fields.	Page 7, 16	KS
		Clarification of primary endpoint according to Phoenix criteria with included reference.	Page 7, 16	KS
		Addition of AE grade 3 or higher including causality assessment for all patients.	Page 29	KS
6.0<br><br>Standard treatment: ADT time restricted for all patients. Salvage RT post prostatectomy is allowed at the discretion of treating physician. Withdrawal of Ad. IV and blood biomarker-collaborator iCellate AB	2024-03-18	Synopsis-standard treatment	Page 7	KS
		Background, ref 17	Page 14	KS
		Overview of study treatment	Page 15	KS
		Study procedures (screening, baseline, EoS)	Page 17-18	KS
		Standard treatment	Page 18-19	KS
		Treatment details spinal lesions, ref added	Page 26	KS
		Safety reporting-clarification of AE/SAE reporting	Page 29	KS
		Blood biomarker	Page 31	KS
6.1	2024-04-22	Ch. 12 SBRT- addition of 0-5 mm CTV-margin including ref	Page 20-27	KS
		Clarification of primary endpoint for recurrent patients post prostatectomy with included reference.	Page 14	KS
6.2	2025-11-10	Minor adjustments in synopsis/protocol for readability<br><br>Adjustment in contact list	 <br><br>Page 3-4	KS<br><br><br>KS

		Minor adjustment statistical plan<br><br>Adjusted Addendum III	Page 7, 29<br><br>AD III	OB<br><br>KS, SS
6.3	2026-01-21	Minor text-adjustments to clarify exclusion criteria 2, 3, 5 and 8<br><br>Minor text-adjustments to clarify the general study oversight	P7,17 , 26<br><br>P37-38	KS<br><br>KS
